# Maternal prenatal co-exposure to air pollution and psychological distress shapes the neonatal gut: microbiota-mediated pathways to early neurodevelopment

**DOI:** 10.1080/19490976.2026.2614451

**Published:** 2026-01-13

**Authors:** Pu Yang, Yifei Pei, Yongqi Huang, Mengyuan Dong, Fangming Cui, Shuang Nie, Xuan Zhang, Fenglin Cao

**Affiliations:** a School of Nursing and Rehabilitation, Shandong University, Jinan, People's Republic of China; b Qilu Second Hospital of Shandong University, Jinan, People's Republic of China; c NHC Key Lab of Health Economics and Policy Research, (Shandong University), Jinan, People's Republic of China

**Keywords:** Air pollution, psychological distress, meconium microbiota, infant neurodevelopment, self-organizing map

## Abstract

Early life gut microbiota function as biological sensors for maternal prenatal exposure and play a crucial role in infant neurodevelopment. During pregnancy, air pollution and psychological distress are regarded as general and specific external exposures, respectively; however, the joint influence of these two domains on shaping early life gut microbiota remains unexplored. In this study, 309 mother-infant pairs were recruited from the obstetrics departments of two tertiary hospitals. We collected data on maternal prenatal air pollution exposure and psychological distress, obtained meconium samples within 48 h after birth, and assessed infant neurodevelopmental outcomes using the Ages and Stages Questionnaire-3 at 1, 3, and 6 months postpartum. Maternal prenatal air pollution-psychological distress exposure patterns were identified using a self-organizing map (SOM). The differential features of the meconium microbiota in relation to co-exposure patterns were assessed using multivariate association of linear models. Finally, the mediating role of the meconium microbiota in co-exposure patterns and infant neurodevelopment was analyzed using mediation analysis. We observed that the meconium microbiota at both the phylum and genus levels differed among the three patterns. *Ruminococcus* mediated the relationship between co-exposure patterns and infant neurodevelopment at 3 months of age (IE = 0.181−0.261, *p*_FDR_ < 0.001). These findings support the inclusion of infant gut microbiota within frameworks assessing the risks of maternal prenatal co-exposure to environmental pollution and psychological distress, providing a scientific basis for policymakers to identify intervention targets for high-risk populations.

## 1. Introduction

Microbiome-host interactions in early life play a critical role in shaping lifelong health and disease states.^1^^,^^2^ During the first two years of life, the human gut microbiota changes from a sparse and variable community to a mature, stable, and adult-like ecosystem.^3^ Microbial colonization can influence the development and maintenance of multiple physiological systems, including the immune system, endocrine system, metabolic processes, maturation, and functional integrity of the brain,^4^^,^^5^ and even human health in adulthood.^5^

Recently, the association between gut microbiota and offspring neurodevelopment has garnered increasing attention. Research has shown that microbiota shaping occurs together with neurodevelopmental processes.^6^ The meconium, generated in the fetal gut from the second month of gestation, accumulates various molecules from the maternal–fetal environment^7^ and provides a material interface linked to gut processes. Meanwhile, the central nervous system begins to develop as early as the first month of pregnancy.^8^ This concurrent development shows that the microbiota–gut–brain axis is a key regulator of neurodevelopmental phases. Gut microbiota regulate early life neurodevelopment through neural, immune, and endocrine pathways.^9^ Recent research has revealed that the infant gut microbiota at 1 week, 6 weeks, and 3−6 months after birth are associated with neurodevelopment at 6 months of age,^10^ anxiety and depression at 4 years of age,^11^ and communication ability and fine motor skills at 3 years of age.^12^

The timing and origin of the first microbial colonization in early life remain subjects of considerable debate. Previous studies have detected bacteria in the placenta, umbilical cord, and amniotic fluid,^2^ suggesting that the initial microbial community of infants before birth may be influenced by the intrauterine environment.^13-16^ However, other studies support the “sterile womb” hypothesis,^17^ which posits that the gut microbiome is primarily established during and shortly after birth.^18^ Regardless of whether stable microbial colonization occurs before birth, the meconium microbiome may reflect both transient prenatal exposure and maternal influences at delivery,^19-21^ providing valuable clues for understanding the onset of early microbial colonization and its maternal determinants.

A promising framework for this investigation is the exposome,^22^ which includes overlapping domains: the internal and external exposomes. The external exposome is further divided into general and specific categories.^23^^,^^24^ Compared with internal exposomes (genetic, immunological, and metabolic factors), external exposomes show greater potential for large-scale prevention and intervention during early life and may thus represent a key target for modulating the meconium microbiota and promoting favorable health outcomes. In this study, we focused on two representative, prevalent, and modifiable prenatal external exposures-air pollution and maternal psychological distress-which reflect the influence of physical and psychosocial environments on fetal development. Air pollution represents general external exposure,^25^ which is physical and quantifiable and can influence fetal development through oxidative stress and inflammatory pathways. In contrast, maternal psychological distress is conceptualized as an external exposome,^25^ reflecting the broader psychosocial contexts that shape the intrauterine milieu. Examining both exposures allowed us to capture the multilevel nature of the external exposome and explore how different domains of prenatal exposure collectively contribute to variations in the intrauterine environment and early life biological processes.

Air pollution is ranked as the top environmental hazard, posing a threat to global health^26^; air pollutants include differently sized particulate matter (PM_2.5_ and PM_10_), carbon monoxide (CO), nitrogen dioxide (NO_2_), and sulfur dioxide (SO_2_). These pollutants can traverse the placental barrier and directly access the developing fetal organism.^27-29^ For example, exposure to PM_2.5_ and its particular components has been associated with modifications in the composition, richness, and evenness of the meconium microbiota.^30^

Psychological distress, comprising anxiety, depression, and insomnia, affects the health of both the mother and child and is experienced by approximately one-quarter of pregnant women.^31^ As early as 2015, Zijlmans^32^ discovered that prenatal psychological distress was related to the gut microbiota. Since then, numerous studies have provided evidence supporting the association between prenatal psychological distress and alterations in the gut microbiota.^33-36^ Prenatal psychological distress can disrupt maternal oral, gut, and vaginal microbiomes, potentially facilitating the translocation of bacteria to the intrauterine environment,^17^ which may in turn regulate the meconium microbiota.^37^

Most studies have isolated the effects of one or more air pollutants^7^^,^^30^ or psychological distress^34^^,^^38^; however, in reality, pregnant women are often exposed to complex mixtures of multiple air pollutants and varying degrees of psychological distress. This complex exposure pattern may potentially affect the meconium microbiota far beyond what single or average exposure studies have revealed. However, to the best of our knowledge, no existing studies have simultaneously considered environmental pollutants and psychological distress as co-exposures to explore their relationship with the meconium microbiota. Furthermore, even in studies that include multiple air pollutants or psychological distress indicators, statistical analyzes often reduce these variables to simplistic binary categories (e.g., exposed vs. not exposed)^34^^,^^38^ or explore one factor while controlling the others.^7^^,^^34^ Such approaches fail to consider the influence of exposure severity and, more importantly, do not fully consider the complex joint effects of different air pollutants or psychological distresses within the exposure system. Therefore, there is an urgent need to integrate high-dimensional multi-exposure omics variables to systematically investigate how prenatal co-exposure patterns shape meconium microbiota. This will allow us to reveal the true biological effects of co-exposure and identify key targets for early life interventions aimed at preventing neurodevelopmental disorders. The first aim of this study was to explore the association between maternal prenatal co-exposure to air pollution and psychological distress patterns and meconium microbiota.

In addition, we aimed to examine the mediating role of the meconium microbiota in the relationship between prenatal co-exposure patterns and infant neurodevelopment. Although several studies have explored the associations among prenatal air pollution, psychological distress, meconium microbiota and early neurodevelopmental outcomes,^7^^,^^30^^,^^34^ they commonly fail to consider multi-omics co-exposures. Addressing this gap is crucial for elucidating the true interactive effects of complex exposures, thus enabling the early identification of populations at high risk of neurodevelopmental disorders and microbiota-targeted intervention strategies.

## 2. Methods

### 2.1. Study design and population

This longitudinal study was conducted from 2020 to 2021 in prenatal clinics located in two large tertiary hospitals in Jinan, Shandong Province, China. The eligibility criteria for participation were as follows: (i) individuals aged ≥ 18 years, (ii) those at 12–20 weeks of gestation, (iii) those with a singleton pregnancy, and (iv) those with adequate literacy in Chinese. Individuals with severe physical or mental illnesses were excluded from the study. This study was approved by the Ethics Committee of the School of Nursing and Rehabilitation, Shandong University (Approval Number: 2020-R-025). We provided informed consent for participation in the study.

### 2.2. Air pollution exposure

Air pollution exposure data sourced from the China High Air Pollutants (CHAP) dataset (Jing Wei) represent a comprehensive collection of long-term, high-resolution, and superior-quality datasets pertaining to ground-level air pollutants across China. The dataset was generated using large data sources. By leveraging artificial intelligence techniques, the dataset accounts for the spatiotemporal variability inherent in air pollution, thereby ensuring accuracy and reliability. The latitude and longitude coordinates were obtained based on the address information, and pregnancy duration was calculated by subtracting the gestational age from the delivery date. Subsequently, we retrieved daily air pollution data at a resolution of 1 km for each pregnant woman from the CHAP database, spanning from the first day of pregnancy until delivery. The data for PM_2.5_, PM_10_, CO, NO_2_, and SO_2_ were averaged over the pregnancy period to obtain air pollution exposure levels for each individual.

### 2.3. Psychological distress exposure

Maternal psychological distress exposure data were prospectively collected during the first, second, and third trimesters (at approximately 16, 24, and 36 weeks of gestation). The Generalized Anxiety Disorder-7 (GAD-7)^39^ scale was used to evaluate the severity of anxiety symptoms. The Edinburgh Postnatal Depression Scale (EPDS)^40^ was used to assess the severity of the depressive symptoms. The Insomnia Severity Index (ISI)^41^ was used to evaluate insomnia severity. These three variables were treated as continuous variables and their values at the three time points were averaged to represent psychological distress.

### 2.4. Meconium microbiota

Within 48 h of birth, 5–6 g of the meconium was collected in tubes and stored in a refrigerator. Subsequently, within 24 h, the researcher transported it to the laboratory and stored it at –80 °C until the sequencing procedure was performed. The methodologies employed for DNA extraction, PCR amplification, Illumina MiSeq sequencing, and bioinformatics analysis, as well as the contamination control measures taken during microbiota analysis, are presented in Appendix A.

### 2.5. Infant neurodevelopment

We conducted follow-ups at time points T4, T5, and T6 (1, 3, and 6 months postpartum) and collected infant neurodevelopmental data using the Ages and Stages Questionnaire-3 (ASQ-3), which contains the domains of gross motor ability, fine motor ability, problem-solving ability, communication, and personal and social ability.^42^ For each domain, an assessment was conducted using six questions designed to determine the attainment of the pertinent skills. A higher score indicated a more advanced level of development.

### 2.6. Covariates

According to previous studies, a series of factors thought to be related to prenatal exposure, infant neurodevelopment, and meconium microbiota were identified as covariates,^7^^,^^30^ such as maternal age, maternal education level (below bachelor, bachelor or above), average monthly household income (<6000 yuan, ≥6000 yuan), pre-pregnancy body mass index, weight gain during pregnancy, smoking during pregnancy (yes or no), drinking during pregnancy (yes or no), frequency of intake of vegetables and fruits,^3^^,^^43^^,^^44^ frequency of intake of high-quality protein, ^3^^,^^44^^,^^45^ and fetal feces collection time. Maternal pregnancy-related characteristics included complications during pregnancy, Group B Strep (yes or no), and vaginitis (yes or no). Delivery-related characteristics included delivery mode (vaginal or cesarean section), infant sex (male or female), infant gestational age at birth, and infant birth weight.^11^ Maternal pregnancy and delivery-related characteristics were obtained from medical records. Other data regarding pregnant women were self-reported at baseline. The definitions of some covariates are presented in Appendix A.

### 2.7. Statistical analysis

#### 2.7.1. Self-organizing map

In this study, the mice package in R was used to conduct multiple imputations of missing covariates using a random forest. Self-organizing map (SOM)^46^ was used to explore prenatal air pollution and psychological distress exposure patterns. Each pattern was named based on its distinct features. A nightingale rose diagram was used to visualize clustering.

#### 2.7.2. Multivariate linear regression and multivariate association of linear models

Multivariate linear regression and multivariate association of linear models was used to explore the correlations among co-exposure patterns, *α* diversity (Chao1, Shannon, Simpson) and meconium microbiota, and infant neurodevelopment (the total score and each dimension of ASQ at three time points).

#### 2.7.3. Principal coordinate analysis, permutational multivariate analysis of variance, and linear discriminant analysis effect size

To compare gut microbiota differences among co-exposure patterns, we performed principal coordinate analysis (PCoA) for visualization, permutational multivariate analysis of variance (PERMANOVA) with post‑hoc testing to assess *β* diversity significance, and linear discriminant analysis effect size (LEfSe) to identify differentially abundant microbial taxa.

#### 2.7.4. Mediation analysis

To investigate whether co-exposure patterns affect infant neurodevelopment through meconium microbiota, we conducted a mediation analysis. Different meconium microbiota and *α* diversity were used as mediating variables, the co-exposure patterns identified by SOM as the independent variable, and the transformed infant neurodevelopment variables as the dependent variable for mediation analysis.

#### 2.7.5. KEGG pathways enrichment analyzes

We predicted the metabolic functional potential of the microbial community by referencing the Kyoto Encyclopedia of Genes and Genomes (KEGG) database to elucidate functional differences among the co-exposure patterns.

#### 2.7.6. Sensitive and additional analyzes

We conducted two sensitivity analyzes. Firstly, we reduced the number of covariates in the MaAsLin and the mediation analyzes. Secondly, we conducted SOM clustering, MaAsLin, and mediation analyzes, excluding 76 participants with pregnancy complications, and using the remaining 233 participants.

We conducted several additional analyzes. Firstly, considering the effect of fluctuations in exposure and trimester-specific levels on infant neurodevelopment, we employed 8 standard deviations and 24 variables for SOM clustering, followed by MaAsLin and mediation analyzes. Secondly, to compare which type of exposures had a greater impact on meconium microbiota and infant neurodevelopment, we used eXtreme gradient boosting (XGBoost)^47^ to rank the importance of five air pollution and three psychological distress indicators for meconium microbiota and infant neurodevelopment. Thirdly, we extracted the principal components from five air pollution and three psychological distress indicators and performed interaction analyzes to examine their joint effects. Fourthly, quantile-based g-computation (qgcomp)^48^ and Bayesian kernel machine regression (BKMR)^49^ were used to further explore mixed-exposure effects.

Psychological distress indicators and Chao1 richness showed right-skewed distributions, infant neurodevelopment indicators and Simpson diversity showed left-skewed distributions, and air pollution indicators showed approximately normal distributions. Right-skewed data were log-transformed as x' = log (x + c), where c = max (0, 1–min(x)). Left-skewed data were transformed as x = x^2^ within the range of 0−10, and data with negative values or a range > 10 were transformed as x' = log (M-x + 1), M = max(x) transformation. The meconium microbiota data were transformed as x' = log (x + 1).

The statistical analyzes were performed using MATLAB R2024b and R 4.4.1. Statistical significance was defined using a two-tailed test with *p* < 0.05. SOM clustering analysis was performed in MATLAB using the SOM toolbox 2.0. R packages in this study included “mice,” “vegan,” “Maaslin2,” “mediation,” “qgcomp,” and “bkmr,” “ggplot2.” The *p-values* were adjusted for multiple testing using the Benjamini-Hochberg false discovery rate (FDR) procedure or the Bonferroni method. The details of all the statistical analyzes are provided in Appendix A.

## 3. Results

A total of 465 pregnant women were included in this study. Of these, 381 provided their home addresses. We collected the delivery dates of 356 individuals with a gestational age of 361. However, only 343 individuals had both home addresses and pregnancy durations (calculated by subtracting the gestational age from the delivery date), which allowed us to obtain environmental data during pregnancy. Finally, we obtained air pollution data for PM_2.5_, PM_10_, CO, NO_2_, and SO_2_ during pregnancy for 343 pregnant women. Among them, 309 provided meconium microbiota samples of infants collected within 2 days after birth, and psychological distress was collected at three time points. Therefore, for the first aim, we used data from 309 mother-infant pairs for analysis.

For the second aim, we conducted follow-ups at time points T4, T5, and T6 (1, 3, and 6 months postpartum) and collected the sample sizes of the infants’ ASQ-3 at 277, 274, and 264, respectively. The characteristics of the participants, as well as descriptions of air pollution, psychological distress, and infant neurodevelopment are shown in Table 1. Mean maternal age was 30.45 years. Of the pregnant women, 65.0% had an education level of bachelor's degree or above, 75.1% of the families had an average monthly income of ≥ 6000 yuan, the average gestational age at birth was 39 weeks, and the average birth weight was 3459.13 g.

**Table 1.** The characteristics of the study population, air pollution, psychological distress, and infant neurodevelopment (*N* = 309, the sample sizes of the infants' ASQ-3 at T4, T5, and T6 time points were 277, 274, and 264, respectively).

Characteristics	**N* (%)/M* ± *SD*/median [IQR]
**Maternal age, year**	30.45 ± 3.62
**Maternal education level**	
Below bachelor	108(35.0%)
Bachelor or above	201(65.0%)
**Average monthly household income, yuan**	
<6000	77(24.9%)
≥6000	232(75.1%)
**Pre-pregnancy body mass index (BMI), kg/m** ^**2**^	23.20 ± 3.69
**Weight gain during pregnancy at baseline, kg**	3.02 ± 4.29
**Complications during pregnancy**	
No	254(82.2%)
Yes	55(17.8%)
**Group B streptococci**	
No	300(97.1%)
Yes	9(2.9%)
**Vaginitis**	
No	298(96.4%)
Yes	11(3.6%)
**Delivery mode**	
Vaginal	161(52.1%)
Cesarean	148(47.9%)
**Smoking during pregnancy**	
No	306(99.0%)
Yes	3(1.0%)
**Drinking during pregnancy**	
No	299(96.8%)
Yes	10(3.2%)
**Frequency of intake of vegetables and fruits**	9.59 ± 0.88
**Frequency of intake of high-quality protein**	14.13 ± 2.94
**Infant gender**	
Male	176(57.0%)
Female	133(43.0%)
**Infant gestational age at birth, weeks**	39.00 [38.00,39.00]
**Infant birth weight, g**	3459.13 ± 415.424
**Fetal feces collection time, hours**	6.96 [3.62,12.08]
**PM2.5, μg/m** ^**3**^	44.38 ± 5.51
T1PM_2.5_	38.01 ± 9.46
T2PM_2.5_	44.84 ± 14.71
T3PM_2.5_	52.9 ± 13.83
**PM10, μg/m** ^**3**^	90.23 ± 10.75
T1PM_10_	79.53 ± 14.43
T2PM_10_	87.28 ± 22.93
T3PM_10_	108.62 ± 24.93
**CO, mg/m** ^**3**^	0.93 ± 0.08
T1CO	0.84 ± 0.11
T2CO	0.96 ± 0.18
T3CO	1.02 ± 0.18
**NO2, μg/m** ^**3**^	39.12 ± 5.18
T1NO_2_	33.68 ± 7.52
T2NO_2_	40.71 ± 11.74
T3NO_2_	45.02 ± 9.13
**SO2, μg/m** ^**3**^	13.23 ± 1.59
T1SO_2_	11.5 ± 1.86
T2SO_2_	13.46 ± 3.77
T3SO_2_	15.43 ± 3.79
**GAD**	3.00 [1.33,4.67]
T1GAD	2.00 [0.00,4.00]
T2GAD	4.00 [1.00,6.00]
T3GAD	3.00 [1.00,6.00]
**EPDS**	4.33 [2.00,7.33]
T1EPDS	4.00 [2.00,7.00]
T2EPDS	4.00 [1.00,8.00]
T3EPDS	4.00 [1.00,8.00]
**ISI**	4.33 [2.00,6.67]
T1ISI	3.00 [1.00,6.00]
T2ISI	4.00 [2.00,7.00]
T3ISI	5.00 [2.00,8.00]
**T4ASQ-3**	245.00 [205.00,270.00]
**T5ASQ-3**	245.00 [205.00,275.00]
**T6ASQ-3**	245.00 [216.25,275.00]

T1, pregnancy trimester 1; T2, pregnancy trimester 2; T3, pregnancy trimester 3; T4, 1 months postpartum; T5, 3 months postpartum; T6, 6 months postpartum.

### 3.1. Maternal prenatal co-exposure patterns identified by SOM

SOM combined with k-means clustering was used to classify and visualize the average daily PM_2.5_, PM_10_, CO, NO_2_, and SO_2_ levels during pregnancy as well as the anxiety, depression, and insomnia values during the first, second, and third trimesters. A final map node matrix of 8 × 10 neurons was applied to describe the similarity between neurons, and the quality evaluation of the map sizes for each category is shown in Table S1. As shown in Figure S1**,** PM_2.5_, PM_10_, CO, NO_2_, and SO_2_ showed similar color distributions, whereas GAD, EPDS, and ISI exhibited similar color distributions, indicating that the five air pollution and three psychological distress indicators were highly correlated, respectively. Three clusters corresponded to an inflection point in elbow graph. Two clusters corresponded to the maximum SC index. The CH index was higher for clusters 2 and 3, with similar values for both clusters. Three clusters corresponded to the minimum DB index (Figure S2). Considering all four indices, we determined that three was the optimal number of clusters.

**Figure 1.**
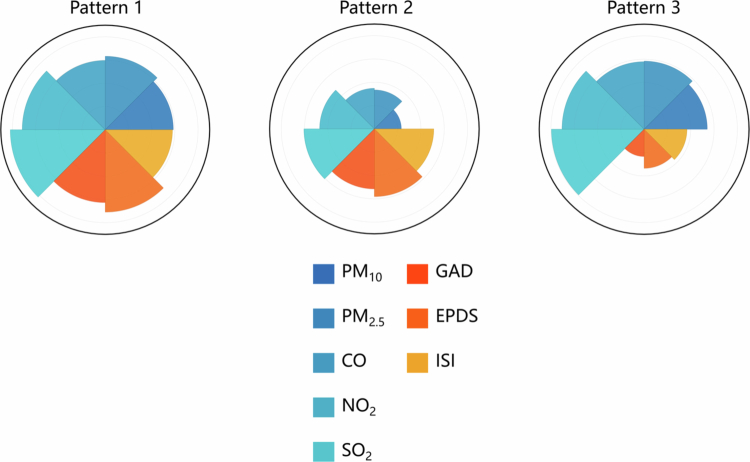
Nightingale rose diagram for the visualization of maternal prenatal co-exposure patterns.

The standardized values of air pollution and psychological distress for each pattern are presented in Table S2, and inter-pattern comparisons of air pollution and psychological distress variables are presented in Figure S3. As shown in Figure 1, we used a nightingale rose diagram to visualize the patterns and highlight the differences between them. Women in pattern 1 experienced the highest air pollution with the highest psychological distress, those in pattern 2 experienced low pollution with moderate psychological distress, and those in pattern 3 experienced high pollution with low psychological distress. Each pattern signifies a unique, inseparable combination of exposure states. Because the results based on pattern 3 as the reference group were not significant or similar to those based on pattern 2 in the subsequent analysis, we mainly present the results based on pattern 2 as the reference group. The results of the multiple linear regression, Masslin, and mediation analyzes conducted using pattern 3 as the reference group are presented in the Appendix B (Table S5, Table S7, Table S9, Figure S7).

### 3.2. Maternal prenatal co-exposure patterns and meconium microbiota

**Figure 2.**
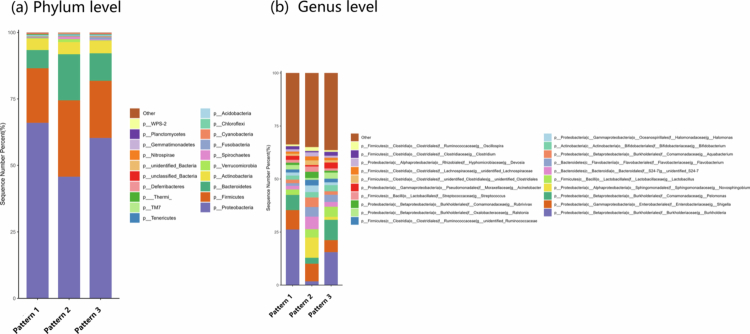
Percentage column stacked chart of different patterns.

**Figure 3.**
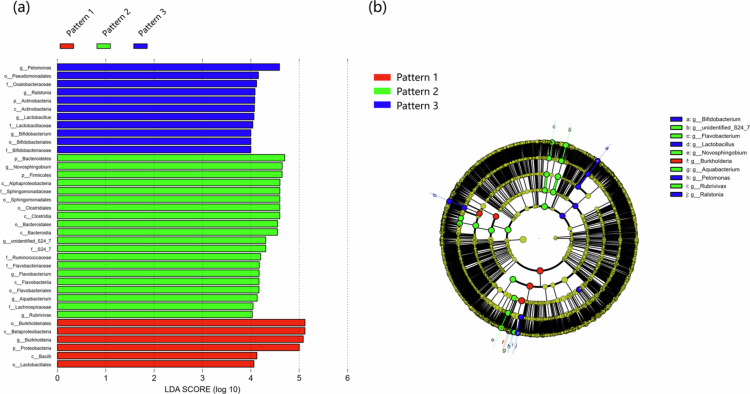
LEfSe multilevel discriminant analysis of species differences. (a) LEfSe analysis histogram. (b) LEfSe analysis phylogenetic tree.

**Figure 4.**
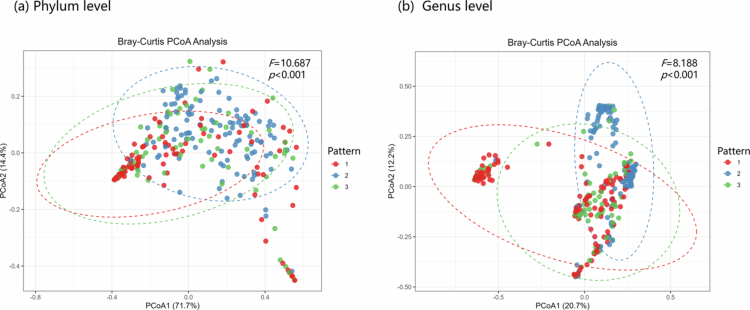
The inter-pattern comparison of *β* diversity.

A percentage column stacked chart of the different patterns at the phylum and genus levels of the meconium microbiota is shown in Figure 2. Furthermore, the differences in the meconium microbiota among the three patterns were analyzed using LEfSe. Significant differences were observed in the abundances of the 37 microbial groups (Figure 3a). The phylogenetic tree is shown in Figure 3b. The three patterns were incorporated into the multivariate linear regression model to explore the differences in meconium microbiota *α* diversity. After conducting multiple test corrections, we found no significant correlations between the variables (Table S4). As shown in Figure 4, when comparing the *β* diversity among the three patterns, significant differences were found at both the phylum (*F* = 10.687, *p* < 0.001) and genus (*F* = 8.188, *p* < 0.001) levels. The pairwise comparisons of *β* diversity are presented in Table S3. We found significant differences regardless of whether the analysis was conducted at the phylum or genus level.

**Figure 5.**
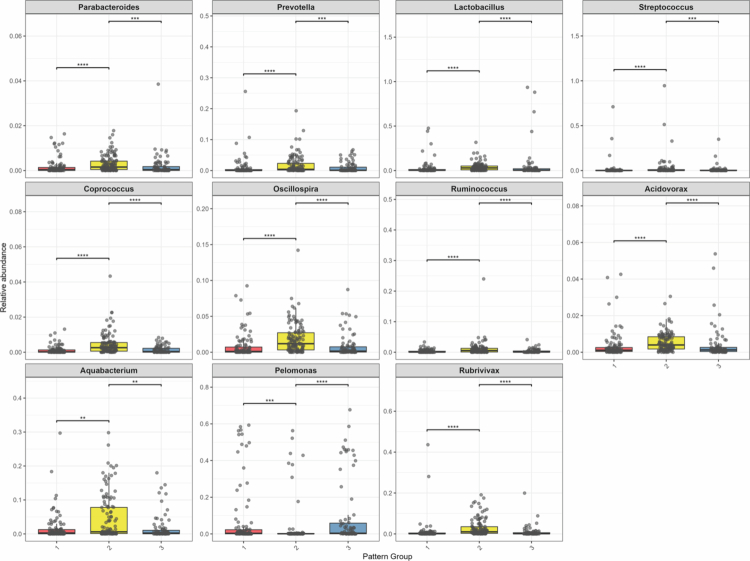
Inter-pattern comparison of meconium microbiota by MaAsLin, except for undefined or unclassified meconium microbiota. * < 0.05; ** < 0.01; *** < 0.001.

In the MaAsLin analysis (Table S8), compared to women in pattern 2 (low air pollution with moderate psychological distress), those in patterns 1 (the highest air pollution with the highest psychological distress) and 3 (high air pollution with low psychological distress) experienced more severe air pollution exposure. Regardless of whether the psychological distress was high (pattern 1) or low (pattern 3), the relative abundance of p_*Bacteroidetes* (including g_*Parabacteroides* and g_*Prevotella*) decreased. At the p_*Firmicutes* phylum level, the relative abundances of g_*Lactobacillus,* g_*Coprococcus*, g_*Oscillospira*, and g_*Ruminococcus*, were comparatively low. At the p_*Proteobacteria* phylum level, the relative abundances of g_*Acidovorax*, g_*Aquabacterium*, and g_*Rubrivivax* were comparatively low. Compared to women in pattern 2 (low air pollution with moderate psychological distress), those in pattern 1 (the highest air pollution with the highest psychological distress) experienced more severe air pollution and psychological distress, and the relative abundance of p*_Firmicutes* (g*_Streptococcus*) was lower. Compared to women in pattern 2 (low air pollution with moderate psychological distress), those in pattern 3 (high air pollution with low psychological distress) had the opposite characteristics, and the relative abundance of g*_Pelomonas* (belonging to p*_Proteobacteria*) was higher. An inter-pattern comparison of the 11 differentially identified meconium microbiota is shown in Figure 5.

### 3.3. Maternal prenatal co-exposure patterns and infant neurodevelopment

The three patterns were incorporated into a multivariate linear regression model to explore the differences in infant neurodevelopment (the total score and each dimension of the ASQ at the three time points), as shown in Table S4. Infant neurodevelopment indicators showed a negatively skewed distribution, which was transformed to x' = log (M-x + 1), M = max(x). Therefore, the subsequent effects were contrary to the original trends observed during neural development. We found that, compared to the infants' mothers in pattern 2 (low air pollution with moderate psychological distress), those in pattern 1 (the highest air pollution with the highest psychological distress) experienced the highest levels of air pollution and psychological distress. The T5 ASQ total score (*B* = 0.450, *SE* = 0.194, *p* = 0.021, *p*_FDR_ = 0.301) and T5 personal-social ability (*B* = 0.603, *SE* = 0.177, *p* = 0.001, *p*_FDR_ = 0.081) of infants were relatively worse in pattern 1. Compared to the infants' mothers in pattern 2 (low air pollution with moderate psychological distress), those in pattern 3 (high air pollution with low psychological distress) experienced higher levels of air pollution and lower levels of psychological distress, the T4 ASQ total score (*B* = –0.357, *SE* = 0.170, *p* = 0.037, *p*_FDR_ = 0.390), T4 gross motor ability (*B* = –0.697, *SE* = 0.211, *p* = 0.001, *p*_FDR_ = 0.081), T4 fine motor ability (*B* = –0.454, *SE* = 0.173, *p* = 0.009, *p*_FDR_ = 0.202), T4 problem-solving ability (*B* = –0.517, *SE* = 0.204, *p* = 0.012, *p*_FDR_ = 0.226), and T6 fine motor ability (*B* = –0.625, *SE* = 0.223, *p* = 0.005, *p*_FDR_ = 0.194) of infants were better in pattern 3. Notably, after the FDR correction, none of the findings were statistically significant. The FDR-corrected significant regression results for the meconium microbiota obtained via MaAsLin analysis and infant neurodevelopment are presented in Table S6. We identified negative correlations between g_*Ruminococcus* and T5 ASQ total score (*B* = –104.533, *SE* = 22.393, *p* < 0.001, *p*_FDR_ = 0.001), T6 ASQ total score (*B* = –40.055, *SE* = 10.876, *p* < 0.001, *p*_FDR_ = 0.022), T5 Fine motor ability (*B* = –108.085, *SE* = 22.345, *p* < 0.001, *p*_FDR_ < 0.001), and T5 Personal and social ability (*B* = –104.019, *SE* = 20.707, *p* < 0.001, *p*_FDR_ < 0.001).

### 3.4. The mediating role of meconium microbiota in the associations between maternal prenatal co-exposure patterns and infant neurodevelopment

**Figure 6.**
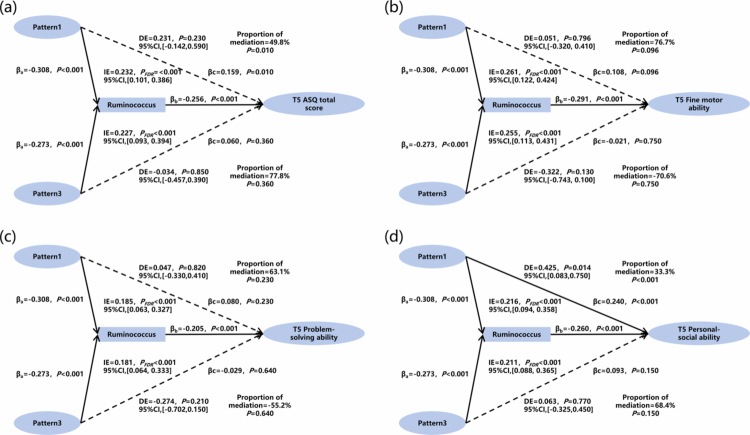
The mediating role of meconium microbiota in maternal prenatal co-exposure patterns and the infant neurodevelopment, pattern 2 was set as the reference. We adjusted for maternal age, maternal education level, average monthly household income, pre-pregnancy body mass index, weight gain during pregnancy, complications during pregnancy, smoking during pregnancy, drinking during pregnancy, infant sex, delivery mode, infant gestational age at birth, infant birth weight, group B streptococci, vaginitis, frequency of intake of vegetables and fruits, frequency of intake of high-quality protein, and fetal feces collection time.

The mediation analysis revealed that, compared with women in pattern 2, g*_Ruminococcus* played a mediating role in the relationships between those in patterns 1 and 3 and the T5 ASQ total score (IE = 0.227−0.232, *p*_FDR_ < 0.001), T5 Fine motor ability (IE = 0.255−0.261, *p*_FDR_ < 0.001), T5 Problem-solving ability (IE = 0.181−0.185, *p*_FDR_ < 0.001), and T5 Personal-social ability (IE = 0.211−0.216, *p*_FDR_ < 0.001) of the infants (Figure 6). The post-hoc power analysis revealed that the impact of all indirect effects exceeded 85% (Table S10).

### 3.5. The predictive analysis of the biological functions of the meconium microbiota associated with the prenatal co-exposure patterns

**Figure 7.**
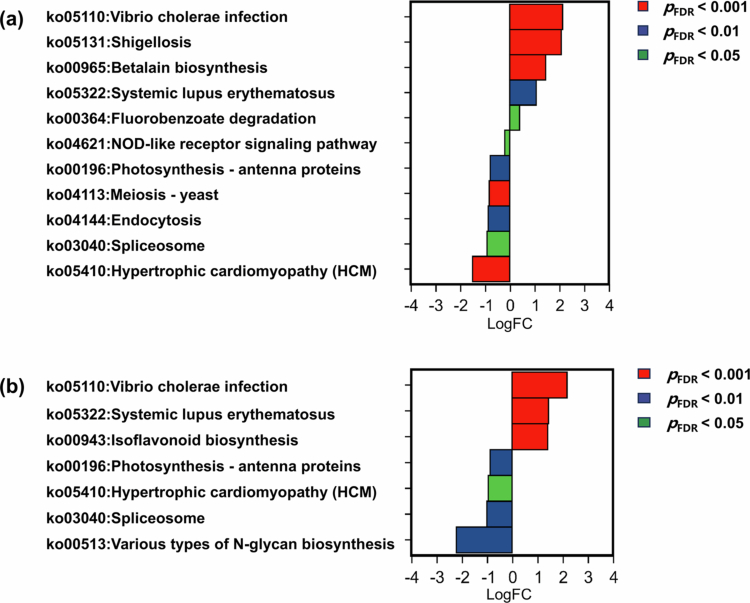
Differential analysis of KEGG pathways of meconium microbiota in maternal prenatal co-exposure patterns. (a) Differential KEGG pathways in the meconium microbiota between patterns 1 and 2. (b) Differential KEGG pathways in meconium microbiota between patterns 3 and 2.

KEGG pathway analysis revealed distinct metabolic profiles in the meconium microbiota of infants born to mothers with different prenatal co-exposure patterns. There were 11 significantly different pathways in the meconium microbiota between patterns 1 and 2, and 7 significant pathways between patterns 3 and 2 (Figure 7). After stringent filtering (*p*_FDR_ < 0.001), seven differentially abundant core pathways were identified across the three exposure patterns. When pattern 2 was set as the reference, ko05110 (vibrio cholerae infection) was significantly upregulated in the meconium microbiota in patterns 1 and 3. The ko05131 (shigellosis) and ko00965 (betalain biosynthesis) were significantly upregulated, while ko04113 (meiosis—yeast) and ko05410 (hypertrophic cardiomyopathy, HCM) were significantly downregulated in the meconium microbiota in pattern 1. The ko05322 (systemic lupus erythematosus) and ko00943 (isoflavonoid biosynthesis) were significantly upregulated in the meconium microbiota of pattern 3. These results indicated that the meconium microbiota associated with different prenatal co-exposure patterns exhibited distinct metabolic profiles.

### 3.6. Sensitivity and additional analysis

The results of sensitivity and the first additional analyzes were consistent with the main results. We found that g_*Ruminococcus* mediated the relationship between maternal prenatal co-exposure patterns and infant neurodevelopment (Figure S5, Figure S6), further confirming the robustness of our findings.

We then used XGBoost to rank the importance of the five air pollutants and three psychological distress indicators for meconium microbiota and infant development and found that air pollution had a greater impact on both the meconium microbiota and infant neurodevelopment (Figure S8). Analysis of the interaction between air pollution and psychological distress on the meconium microbiota and infant neurodevelopment revealed that when maternal prenatal psychological distress was more severe, the slope of higher air pollution levels and lower relative abundance of g_*Ruminococcus* was almost flat. When maternal prenatal psychological distress was more severe, the slope of higher air pollution and poorer personal-social skills of infants at 3 months was steeper (Figure S9). Notably, after the FDR correction, none of the findings were statistically significant.

The results of the mixed exposure analysis conducted using the qgcomp and BKMR models are shown in Figure S10. In the qgcomp model, the overall effect of the maternal prenatal co-exposure was positively correlated with the transformed T5 ASQ total score (*ψ* = 0.340, *p* = 0.012) and negatively correlated with g_*Ruminococcus* (*ψ* = –0.005, *p* = 0.003). Air pollution consistently had the largest weight associated with all outcomes. In the BKMR model, the exposure-response relationships all showed approximately linear patterns.

## 4. Discussion

The current study identified three maternal prenatal co-exposure patterns based on SOM. Furthermore, we explored the differences in meconium microbiota composition across these patterns and found that the meconium microbiota mediated the relationship between maternal prenatal co-exposure patterns and infant neurodevelopment. Significant differences in the meconium microbiota were observed at both the phylum and genus levels among the three patterns. Compared with pattern 2, g_*Ruminococcus* mediated the relationships between patterns 1 and 3 and the ASQ total score, fine motor ability, problem-solving ability, and personal-social ability at 3 months. In terms of variable importance and weight map, maternal prenatal air pollution exposure showed a stronger correlation with both meconium microbiota and infant neurodevelopment than maternal prenatal psychological distress. These findings provide the latest scientific evidence for the origin of the development of the gut microbiota-brain axis and its connection to important early neurodevelopmental characteristics.

### 4.1. The association between maternal prenatal co-exposure patterns and meconium microbiota

In the MaAsLin analysis, women in patterns 1 (highest air pollution with the highest psychological distress) and 3 (high air pollution with low psychological distress) experienced more severe air pollution exposure than women in pattern 2 (low air pollution with moderate psychological distress). Regardless of whether the psychological distress was high (pattern 1) or low (pattern 3), the relative abundance of p_*Bacteroidetes* (including g_*Parabacteroides* and g_*Prevotella*) decreased. At the p_*Firmicutes* phylum level, the relative abundances of g_*Lactobacillus,* g_*Coprococcus*, g_*Oscillospira*, and g_*Ruminococcus*, were comparatively low. At the p_*Proteobacteria* phylum level, the relative abundances of g_*Acidovorax*, g_*Aquabacterium*, and g_*Rubrivivax* were comparatively low. These findings are consistent with those of previous studies. Previous studies have revealed that prenatal psychological distress is negatively correlated with p_*Proteobacteria,*^50^ g*_Ruminococcus,*^51^ and g*_Lactobacillus*^32^^,^^51^ in the infant gut. Similarly, prenatal air pollution exposure is negatively correlated with g*_Parabacteroides,*^30^^,^^52^ g*_Lactobacillus,*^30^ and *unclassified* f*_Ruminococcaceae*^53^ in the guts of infants and toddlers. Notably, the findings of the current study regarding the relationship between air pollution and g*_Ruminococcus* are contrary to those of previous studies.^52^ Possible reasons include inconsistent measurement times of infant gut microbiota, inconsistent exposure times to air pollution, and differences in various confounding factors. While the current study advanced the time window to 48 h after infant birth, future research still requires extensive studies to explore the association between prenatal exposure and gut microbiota in early life.

### 4.2. The association between maternal prenatal co-exposure patterns and infant neurodevelopment

The current study revealed that, compared to infants whose mothers were exposed to low air pollution and moderate psychological distress (pattern 2), those whose mothers were exposed to high air pollution but low psychological distress (pattern 3) showed better infant neurodevelopment. However, after FDR correction, none of the findings were statistically significant. These findings may suggest that, in environments with high air pollution, low psychological distress may exert a stronger protective effect on infant neurodevelopment, potentially offsetting or even surpassing the detrimental impact of air pollution itself. This protective effect may stem from a context characterized by abundant resources, robust social support, and mothers’ greater coping skills and psychological resilience, which collectively provide substantial buffering and compensatory mechanisms for infant development. Previous studies have demonstrated that mild prenatal psychological distress can accelerate motor, cognitive, and language development, indicating that not all psychological distress exposures are detrimental.^54^^,^^55^

Interaction analyzes revealed that among mothers with high psychological distress, the association between air pollution exposure and infant neurodevelopment (specifically, personal-social abilities at 3 months) were stronger than that among mothers with low psychological distress, although the relationship was no longer significant after FDR correction. This may indicate that psychological distress amplifies the toxic effects of environmental chemicals on developmental outcomes, consistent with the findings of previous studies of neurotoxic substances. ^56-58^

Mechanistically, research has indicated that air pollutants and trace metals adsorbed in air pollution can directly cross the placenta and enter fetal circulation.^28^^,^^29^ This direct passage allows pollutants to affect the fetal brain and disrupt essential metal homeostasis, thereby interfering with normal neural development.^28^ Air pollution also directly compromises placental function,^29^ leading to maternal systemic and placental inflammation and oxidative stress, alterations in placental DNA methylation, and dysregulation of the hypothalamic-pituitary-adrenal (HPA) axis, all of which represent potential pathways influencing neural development.^29^ Psychological distress similarly exerts negative effects on infant neurodevelopment by inducing changes in the filtering capacity of the placenta,^59^ brain structure, and function caused by neuroendocrine and inflammatory processes, epigenetics, the HPA axis, and inflammation.^54^^,^^60^ Therefore, the amplified effects of high psychological distress on air pollution toxicity may stem from their shared ability to act on overlapping biological pathways.

Finally, we used XGBoost to rank the importance of five air pollutants and three psychological distress indicators for the meconium microbiota and infant development, revealing that air pollution had relatively great impact on both outcomes. The weight map obtained using the qgcomp model supported this finding. This may be because air pollution can directly cross the placenta and affect the infant's brain, whereas the cumulative effects of psychological distress may be comparatively less pronounced. Moreover, the objective measurement of air pollutants contrasts with the subjective recall of psychological distress, which may underestimate true exposure. Nonetheless, existing evidence indicates that different prenatal psychological distress indicators can influence the diversity and composition of gut microbiota during the first year of life.^61^ Therefore, the psychological distress indicators used in the current study are of crucial for clarifying the current research topics. This result suggests that compared to reducing psychological distress, reducing pregnant women's exposure to air pollution may be a more direct and effective way of protecting their offspring from neurodevelopment. Future studies should consider the multidimensional nature of psychological distress to capture its actual impact.

### 4.3. The mediating role of meconium microbiota in the association between maternal prenatal co-exposure patterns and infant neurodevelopment

Mediation analysis showed that, compared with women in pattern 2 (low air pollution with moderate psychological distress), g*_Ruminococcus* played a mediating role in the relationships between those in patterns 1 (highest air pollution with highest psychological distress) and 3 (high air pollution with low psychological distress) and a decrease in infant ASQ total score, fine motor ability, problem-solving ability, and personal-social ability at 3 months. These findings are consistent with those of previous studies. For example, one study reported that the abundance of g*_Ruminococcus* in fecal samples collected at 6 weeks of age was negatively correlated with the degree of depressive symptoms at 4 years of age.^11^ Another study found that the g_*Ruminococcus* abundance at 18 months of age was negatively associated with aggression scores at age 3.5.^62^

*Ruminococcus* is an anaerobic bacterium involved in the degradation of complex carbohydrates and production of short-chain fatty acids (SCFA), including butyric, acetic, and propionic acids. These SCFAs are key metabolites of the gut-brain axis and exert regulatory effects through several mechanisms.^63^ For example, butyrate can influence the blood-brain barrier, modulate neurotransmitter levels and neurotrophic factors, and directly stimulate the vagus nerve, affecting brain regions involved in emotional regulation, stress response, and cognition.^64^ SCFAs also inhibit histone deacetylase (HDAC)-mediated gene expression in the brain and play roles in immune regulation (via acetate), anti-inflammatory processes, enhancement of gut barrier integrity, and energy metabolism.^65^^,^^66^ Future research should further investigate the unique role of g*_Ruminococcus* in developmental programming and verify the direct evidence of SCFA regulation on infant neurodevelopment through a repeated measurement design to analyze the impact of dynamic changes in g*_Ruminococcus*. Such insights could support the development of interventions targeting the gut-microbiota-brain axis to promote healthier neurodevelopmental outcomes.

### 4.4. The KEGG differential pathways of meconium microbiota related to the prenatal co-exposure patterns

We predicted the KEGG pathways in the meconium microbiota across the different co-exposure patterns. Compared to the meconium microbiota of infants born to women in pattern 2, the pathway ko05110 (vibrio cholerae infection) was significantly upregulated in the meconium microbiota of infants born to women in patterns 1 and 3. Previous studies have demonstrated an antagonistic interaction between *Ruminococcus obeum* and *Vibrio cholerae*. In co-colonized mice, *R. obeum* inhibited the expression of multiple virulence factors and reduced *V. cholerae* colonization^67^; and in household contacts of cholera patients in Bangladesh, higher *Ruminococcus* abundance was associated with resistance to infection.^68^^,^^69^ Notably, we observed reduced g*_Ruminococcus* abundance in the meconium microbiota in patterns 1 and 3, along with the enrichment of ko05110, suggesting that the depletion of *Ruminococcus* may compromise colonization resistance and correlate with enhanced potential pathogen activity. However, such dysbiosis likely arises from complex host-microbe-environment interactions rather than direct causation. Furthermore, we found significant upregulation of ko00965 (betalain biosynthesis) in the meconium microbiota in pattern 1. A study reported that the consumption of red beetroot, a rich source of betalains, was negatively correlated with *Ruminococcus* abundance.^70^ Additionally, in the meconium microbiota of infants born to women in pattern 3, we observed significant upregulation of ko05322 (systemic lupus erythematosus). A previous study found the depletion of an unclassified bacterium within the f*-Ruminococcaceae* in patients with systemic lupus erythematosus,^71^ which aligns with our observation of reduced *Ruminococcus* in the meconium microbiota in pattern 3. Briefly, our findings revealed associations between distinct prenatal co-exposure patterns and the enrichment of specific functional pathways, along with a reduced abundance of commensal taxa, such as *Ruminococcus*. These results suggest that prenatal environments may shape the functional potential of early life gut microbiota, potentially influencing disease susceptibility. However, these associations require validation in larger cohorts and further mechanistic exploration using multi-omics approaches such as metagenomics and metabolomics.

In summary, the present study proposes an innovative risk assessment framework that elucidates how prenatal environmental exposure and maternal psychological distress may influence offspring neurodevelopment via alterations in early life gut microbiota. This integrative perspective highlights the gut microbiome as a potential biomarker for assessing the cumulative effects of complex co-exposures, advancing public health research beyond single-factor evaluations to a holistic, system-based risk assessment paradigm. By identifying distinct exposure profiles, the framework supports the stratification of vulnerable mother–infant dyads, offering a foundation for precision prevention and timely public health interventions. Moreover, the discovery of specific microbial mediators, such as g_*Ruminococcus*, in the exposure–neurodevelopment pathway provides novel microbial targets for low-cost, population-level interventions, including probiotic, prebiotic, and dietary strategies. Given that the gut microbiota begins to establish during the perinatal period and is associated with neurodevelopmental outcomes as early as 3 months of age, these findings emphasize the critical early window for public health action to promote long-term neurodevelopmental health.

The current study has the following advantages. First, by integrating SOM to classify the co-exposure patterns of air pollution and psychological distress, this study differs from traditional single-exposure models and emphasizes the importance of non-physical exposure as a potential modulator of physical environmental risks. Second, this study incorporated exposome and microbiome data to provide a comprehensive view of the biological mechanisms involved. Third, our sample size of the current study was relatively large.

This study has the following limitations. (i) Infant neurodevelopmental results reported by mothers who have experienced psychological distress may deviate from the actual situation. (ii) Meconium microbiota was assessed only once, within 48 h of birth. While the meconium is a valuable marker for prenatal exposure, the absence of longitudinal data limits our understanding of postnatal microbiota development. (iii) Key mediators of the microbiota-gut-brain axis, such as SCFAs, serotonin, and inflammatory markers, were not assessed, thereby limiting insights into the biological pathways involved. (iv) 16S rRNA gene sequencing cannot identify specific taxa at the species level. However, 16S rRNA sequencing is relatively economical and suitable for large-scale studies such as those described in the current study. Metagenomic sequencing should be applied in future studies to further explore the differential enterobacteria and reveal potential pathways. (v) Although this study had a prospective longitudinal design with a clear temporal sequence of maternal prenatal exposure, meconium microbiota, and infant neurodevelopment, making reverse causation unlikely, causal inferences should be made cautiously. Further experimental studies, such as those using animal models or randomized controlled trials, are needed to validate these findings and elucidate the underlying mechanisms. (vi) The current study was conducted in two tertiary hospitals in Jinan, Shandong Province, China; therefore, the findings may have limited generalizability to populations or regions with different environmental exposures or cultural backgrounds. Previous studies have shown that factors such as residence and ethnicity may influence meconium microbiota.^21^ Future studies should replicate and verify these findings in more diverse populations and regions.

## 5. Conclusion

The current study reveals the joint effect of maternal prenatal co-exposure to air pollution and psychological factors on shaping the neonatal gut and infant neurodevelopment. It shows that g*_Ruminococcus* mediates the relationship between prenatal maternal co-exposure patterns and infant neurodevelopment. By innovatively integrating high-dimensional exposure pattern analysis and multiomics data, this study revealed a non-negligible joint effect of co-exposure on outcomes. Although air pollution exposure may not be reduced as quickly and effectively as expected, this study provides a public health case study for reducing human exposure to air pollution and psychological distress. Importantly, this study provides strong evidence to support the inclusion of infant gut microbiota in the framework for assessing the risks of environmental pollution and psychological distress, providing an important scientific basis for policymakers in the field of intervention targets for high-risk populations.

## Supplementary Material

Supplementary material Appendix B

## Data Availability

Sequence data is deposited at the NCBI under BioProject repository identifier PRJNA1299033. URL: https://dataview.ncbi.nlm.nih.gov/object/PRJNA1299033?reviewer=iil25038nm0321ffg0hh829c9d. All additional datasets are available from the corresponding author upon reasonable requests.

## Appendix A

### DNA extraction, PCR amplification, Illumina MiSeq sequencing, and Bioinformatic analysis

DNA extraction

Total genomic DNA was extracted using the OMEGA Soil DNA Kit (M5635-02; Omega Bio-Tek, Norcross, GA, USA) according to the manufacturer’s instructions. The quantity and quality of the extracted DNA were measured using a NanoDrop NC2000 spectrophotometer (Thermo Fisher Scientific, Waltham, MA, USA) and agarose gel electrophoresis, respectively.

PCR amplification and Illumina MiSeq sequencing

Polymerase chain reaction (PCR) amplification of the bacterial 16S rRNA gene V3–V4 region was conducted using the forward primer 338F (5’-barcode + ACTCCTACGGGAGGCAGCA-3’) and reverse primer 806 R (5’-GGACTACHVGGGTWTCTAAT-3). Sample-specific 7-bp barcodes were incorporated into the primers for multiplex sequencing. The PCR components comprised 5 μL of 5 × reaction buffer, 5 μL of 5 × FastPfu buffer, 0.25 μL of Fast Pfu DNA polymerase (5 U/μL), 2 μL (2.5 mM) of dNTPs, 1 μL (10 µM) of each forward and reverse primer, 2 μL of the DNA template, and 8.75 μL of ddH2O. Thermal cycling consisted of an initial denaturation at 98 °C for 30 s, followed by 25–27 cycles consisting of denaturation at 98 °C for 15 s, annealing at 50 °C for 30 s, and extension at 72 °C for 30 s, with a final extension of 5 min at 72 °C. PCR amplicons were purified using Vazyme VAHTSTM DNA Clean Beads (Vazyme, Nanjing, China), quantified using the Quant-iT PicoGreen dsDNA Assay Kit (Invitrogen, Carlsbad, CA, USA), and further quantified using QuantiFluor™-ST (Promega, Madison, WI, USA). After individual quantification, the amplicons were pooled in equal amounts, and paired-end 2 × 250 bp sequencing was performed using the Illumina MiSeq platform with MiSeq Reagent Kit v3 at Shanghai Personal Biotechnology Co., Ltd. (Shanghai, China).

Bioinformatic analysis

Microbiome bioinformatics were conducted using QIIME2 with minor modifications according to the official tutorials (https://docs.qiime2.org/2019.4/tutorials/). Briefly, raw sequence data were demultiplexed using the demux plugin, followed by primer cutting with the cutadapt plugin.^72^ Sequences were subsequently quality-filtered, denoised, merged, and chimeras were removed using the DADA2 plugin.^73^ Non-singleton amplicon sequence variants (ASVs) were aligned with mafft^74^ and used to construct a phylogeny with fasttree2.^75^ Taxonomy was assigned to ASVs using a pre-trained naive Bayes classifier in QIIME2, referencing the Greengene database.

### Contamination control measures taken during the microbiota analysis

Negative control samples (CK) were included in each PCR batch to ensure validity. Each CK consisted of sterile water and a PCR master mix, excluding the DNA templates. All CK controls showed no visible bands on agarose gel electrophoresis, confirming the absence of detectable contamination in the reagents or during the PCR setup. As no amplification occurred, the CK samples were not sequenced because they lacked amplifiable products. This standard practice ensured the reliability of the PCR results for the actual samples.

### Definitions of covariates

Gestational diabetes mellitus, hypertensive disorders of pregnancy, and gestational anemia were evaluated as complications during pregnancy. Each complication was coded as present (1) or absent (0), and the total score was calculated to generate a cumulative complication index ranging from 0 to 3.

Smoking and alcohol consumption during pregnancy were initially categorized into four groups: never used, ceased use before pregnancy, occasional use during early pregnancy, and continued use throughout pregnancy, coded from 1to 4, respectively. Participants selecting options 1 or 2 were classified as “no,” whereas those selecting options 3 or 4 were classified as “yes.”

The original frequency categories for the intake of vegetables, fruits, and high-quality protein were daily, more than three times per week, one to three times per week, occasionally, and never, coded from 1 to 5, respectively. After reverse scoring, the combined fruit and vegetable intake was used to compute a cumulative frequency index, with a maximum score of 10. The consumption of milk, eggs, meat/poultry/fish, and legumes was combined to calculate a cumulative index of high-quality protein intake frequency, with a maximum score of 20.

### Details of the statistical analysis

1. Self-organizing map

Self-organizing map (SOM)^46^ was employed to explore co-exposure patterns of prenatal maternal air pollution and psychological distress. SOM is an artificial neural network model that enables multivariate analysis through an unsupervised self-learning mechanism. It is suitable for exploratory data analysis and pattern recognition. SOM is capable of performing non-linear, topology-preserving mapping from ahigh-dimensional input space onto a two-dimensional grid. Topological preservation allows for the clear visualization of global data distribution patterns, potential cluster structures, category transition boundaries, and outliers, thereby significantly reducing the complexity of the initial data exploration. Its interpretability is significantly improved when combined with clustering techniques such as k-means, hierarchical clustering, and principal component analysis.^76^

Prior to analysis, the data were normalized to standardize the values. Based on the principle that the number of map nodes = 5√n (where *n* is the sample size),^76^ the number of map nodes in this study was 87.89. To determine the optimal map node layout, we tested grid configurations of 8 × 10, 9 × 10, and 10 × 10, evaluating the map quality using the quantization error (QE), topographic error (TE), and distribution matching error (DME).^76^ A comprehensive score was calculated by assigning weights of 50%, 30%, and 20% to QE, TE, and DME, respectively. The matrix with the lowest score was selected to map the high-dimensional data of the participants onto a two-dimensional plane. Subsequently, k-means clustering was applied to the SOM node weights. The optimal number of clusters was determined using the elbow plot, Silhouette Coefficient (SC), Calinski-Harabasz index (CH), and Davies-Bouldin index (DB). The inflection point on the elbow plot indicates the optimal number of clusters. The optimal cluster number is further identified by the maximum SC and CH values and the minimum DB value. Finally, each pattern was labeled based on its distinguishing characteristics. The clustering results were visualized using a Nightingale rose chart.

2. Multivariate linear regression and multivariate association of linear models

The prenatal maternal co-exposure patterns were incorporated into the multivariate linear regression model to explore the differences in the meconium microbiota *α* diversity (Chao1, Shannon and Simpson) and infant neurodevelopment (the total score and each dimension of the ASQ at three time points). Multivariate association of linear models (MaAsLin) was used to examine the associations between prenatal maternal co-exposure patterns and the meconium microbiota. False discovery rate (FDR) correction was applied with a threshold of *p*_FDR_ ≤ 0.01. Minimum abundance and prevalence thresholds were 0.0005 and 0.5, respectively. The meconium microbiota identified through MaAsLin were subsequently included in multivariate linear regression models to evaluate their association with infant neurodevelopment.

3. Principal coordinate analysis and permutational multivariate analysis of variance

Principal coordinate analysis (PCoA) was used to visualize sample differences based on the bray-curtis dissimilarity index. To evaluate the statistical significance of *β*-diversity differences among patterns, a permutational multivariate analysis of variance (PERMANOVA) was conducted with 9,999 permutations. A post-hoc pairwise comparison of β-diversity was conducted.

4. Linear discriminant analysis effect size

Linear discriminant analysis effect size (LEfSe) was conducted. The relative abundance values were normalized to a total of one million. The significance level was set to *α* = 0.05, linear discriminant analysis (LDA) effect size at the genus level was > 4, and a strict all-against-all comparison.^77^ LEfSe analysis was conducted using Wekemo Bioincloud (https://www.bioincloud.tech).^78^

5. Mediation analysis

Various meconium microbiota and *α* diversity indices (Chao1, Shannon, Simpson) were used as mediators, exposure patterns identified by SOM served as independent variables, and transformed infant neurodevelopmental measures were used as dependent variables in the mediation analysis. The mediation effect was evaluated using the causal stepwise framework by Baron and Kenny. The significance of the indirect effect was estimated using the bootstrap method (1,000 resamples) with FDR correction, and *p*_FDR_ ≤ 0.05 was considered statistically significant. Post-hoc power analysis was conducted using MedPower to estimate the power of the mediation tests.^79^ Given the sample size and standardized path coefficients (a, b, c'), with *α* = 0.05, we calculated the statistical power for the indirect effect (a × b).

6. KEGG pathways enrichment analyzes

Based on the ASV sequences and abundance tables obtained from 16SrRNA amplicon sequencing, PICRUSt2 was used to predict the microbial functional metabolic potential associated with prenatal co-exposure patterns. Feature sequences with a nearest sequence taxon index (NSTI) > 2 were excluded. Functional pathway abundances were predicted based on the Kyoto Encyclopedia of Genes and Genomes database (KEGG). KEGG KO pathway abundances were normalized as copies per million (CPM). Differentially abundant pathways between groups were identified using a zero-inflated log-normal model in metagenomeSeq (fitFeatureModel function). All analyzes were conducted using GeneCloud, a free web-based platform for data analysis (https://www.genescloud.cn).

7. Sensitive and additional analyzes

We conducted two sensitivity analyzes. Firstly, considering that the sample size was limited and the incorporation of many covariates could lead to overfitting, decreased statistical power, and unstable estimation of parameters. We reduced the number of covariates in the MaAsLin and mediation analysis. In the MaAsLin analysis, adjustments were made for six covariates: maternal age, socioeconomic status, lifestyle during pregnancy, pre-pregnancy BMI, complications during pregnancy, and infant gender. In the mediation analysis, seven covariates were included by adding infant gestational age at birth to the aforementioned variables. Socioeconomic status and lifestyle during pregnancy were newly derived as composite variables in this analysis. Specifically, socioeconomic status was calculated by combining average monthly household income and maternal education level into a composite score ranging from 0 (income < 6,000 yuan and education below a bachelor's degree, indicating lower socioeconomic status) to 2 (income ≥ 6,000 yuan and education at or above a bachelor's degree, indicating a higher socioeconomic status). Lifestyle during pregnancy was calculated by summing smoking and drinking behaviors during pregnancy into a score ranging from 0 (no smoking or drinking, indicating a healthy lifestyle) to 2 (both smoking and drinking, indicating an unhealthy lifestyle).

Secondly, to account for the potential influence of pregnancy complications-including gestational diabetes, gestational hypertension, and gestational anemia, which may substantially affect the composition and abundance of meconium microbiota, we excluded 76 participants with such complications and re-conducted the SOM clustering, MaAsLin, and mediation analyzes using data from the remaining 233 participants.

We conducted several additional analyzes. Firstly, to explore the effects of exposure fluctuation and trimester-specific levels on infant neurodevelopment, we calculated the standard deviation for each of the eight exposure variables. Specifically, for each participant and each exposure variable, three values were used to calculate the standard deviation. For air pollution, these three values represent the average concentrations during three gestational periods (<16 weeks, 16−27 + 6 weeks, and 28−41 weeks); for psychological distress, they correspond to measurements obtained at approximately 16, 24, and 36 gestational weeks. These standard deviations captured intra-individual fluctuations in exposure levels and were used as input features for SOM clustering, followed by MaAsLin and mediation analysis.

Then, we used the average values of the same eight exposure variables across the three gestational windows, resulting in 24 averaged exposure variables per participant (5 air pollutants × 3 periods and 3 psychological distress measures × 3 time points, assessed at approximately 16, 24, and 36 gestational weeks). These 24 variables were used in SOM clustering, and the resulting cluster assignments were incorporated into the subsequent MaAsLin and mediation analyzes.

Secondly, we employed extreme gradient boosting (XGBoost)^47^ to compare the relative contributions of various prenatal exposures, including air pollution and psychological distress, to meconium microbiota composition and infant neurodevelopment. Specifically, five air pollution indicators and three psychological distress indicators were included as predictors. XGBoost ranked the predictors based on their relative importance in explaining the variation in meconium microbiota and infant neurodevelopment.

Thirdly, we extracted the principal components (PCs) from the five air pollutions and three psychological distress indicators, respectively, to represent composite exposure profiles. Interaction analyzes were then performed to evaluate the joint effects of these components. Specifically, we assessed the interactions between PCs and differential meconium microbiota identified via MaAsLin and significant infant neurodevelopmental outcomes identified via mediation analysis. This approach enabled us to investigate whether prenatal air pollution and psychological distress interactively influence the infant meconium microbiota and neurodevelopment.

Fourthly, quantile-based g-computation (qgcomp)^48^ was used to estimate the weights of individual prenatal exposures and the overall effect in the associations with infant neurodevelopment total scores at three time points and the significant meconium microbiota in the mediation analysis. We further employed Bayesian kernel machine regression (BKMR)^49^ to explore the overall and nonlinear effects among the mixed-exposures. BKMR allows flexible modeling of complex exposure-response relationships and interaction effects. For each outcome, analyzes were restricted to individuals with complete data. BKMR estimation was based on 10,000 iterations, ensuring stable posterior inference.

### Reasons for using SOM as the clustering method in current study

An increasing number of methods are now available to visualize similarities and clusters among exposure factors, such as Louvain clustering and Dirichlet multinomial mixture (DMM). After comprehensive consideration, SOM was chosen over these methods for the following reasons.

Louvain clustering, including its variants such as Leiden, has been predominantly applied and validated in domains such as social network analysis, biological networks (e.g., lab indicators,^80^ cell sequencing data,^81^^,^^82^ and cytokine networks,^83^) and medical data.^84^^,^^85^ However, its application in environmental exposure clustering remains limited. Furthermore, the Louvain algorithm fundamentally requires a graph structure. Applying graph-based clustering methods requires converting raw feature vector data into a meaningful graph format. This conversion process is complex and introduces considerable subjectivity and potential bias. Common approaches include using algorithms such as Phenograph,^80^ CoNet, or K-Nearest Neighbors network inference methods,^84^ or employing dimensionality reduction (e.g., PCA) prior to graph construction.^83^ The choice of graph construction method, parameter settings, and connectivity properties of the resulting graph can significantly influence the final clustering results.

DMM is a powerful generative model, primarily suited for modeling discrete data distributions such as microbial community composition^86^ and topic modeling.^87^^,^^88^ Similar to Louvain, its documented use in clustering environmental exposure data (typically involving numerous continuous variables) is extremely limited.

In contrast to Louvain and DMM, SOM has a well-established and extensive record of successful application in environmental data analysis.^76^ The strength of SOM lies in its ability to preserve the topological structure of the original high-dimensional input space within a low-dimensional output map, which is not possible in the case of one of the most widely used k-means algorithms^89^ or DMM and Louvain clustering. This topological preservation facilitates clear visualization of global distribution patterns, underlying cluster structures, category transition boundaries, and discrete outliers, thereby reducing the complexity of early-stage data exploration. Its maturity, interpretability, and suitability for handling high-dimensional continuous variables in environmental research make it a proven and reliable choice for our specific problem. In the current study, the SOM was used as a powerful tool for dimensionality reduction, noise reduction, and data summarization. K-means was then applied to cluster the SOM neurons rather than the raw data. This approach enhances the stability and interpretability of subsequent k-means clustering compared to applying it directly to raw, high-dimensional data.

### Reasons for using the standard deviation of the variables as clustering inputs

Relying solely on average levels of air pollutants throughout pregnancy may obscure fluctuations and their health impacts; therefore, the standard deviation was included to reflect exposure fluctuations. This result aligns with the primary finding that g_*Ruminococcus* continued to mediate the relationship between prenatal co-exposure patterns and infant neurodevelopment. Compared to women in pattern 1 (experiencing high air pollution fluctuations and low psychological distress fluctuations), women in pattern 2 (experiencing low air pollution fluctuations and high psychological distress fluctuations) had lower g_*Ruminococcus* abundance and poorer infant neurodevelopment. These findings further support the robustness of our results when accounting for temporal exposure fluctuations.

The association between standard deviation of variables and health outcomes has been widely studied. One study^90^ used the experience sampling method to collect positive affect (PA) and negative affect (NA) scores from adolescents. Emotional variability was quantified using the standard deviation of each participant’s PA and NA scores. The results indicated that emotional variability significantly predicted depressive symptoms in adolescents. Another study^91^ calculated the standard deviation of temperature and found the association with premature birth. Additionally, two studies^92^^,^^93^ utilized wearable device data to predict health outcomes, incorporating both the mean and standard deviation of variables as predictors. The study found that features capturing variability in sleep patterns were the most predictive, whereas features reflecting average behavior were generally ranked lower.^93^
