# Supplementary material for: Maternal prenatal co-exposure to air pollution and psychological distress shapes the neonatal gut: microbiota-mediated pathways to early neurodevelopment
Source: Gut Microbes. 2026 Jan 13;18(1):2614451. doi: 10.1080/19490976.2026.2614451 (PMC12818825; doi:10.1080/19490976.2026.2614451)
Supplement: Supplementary material — Appendix B [file KGMI_A_2614451_SM1883.docx]

**Appendix B**

[Table S1. Quality evaluation of map sizes for each category 44](#_Toc217142697)

[Table S2. The standardized values of maternal prenatal air pollution and psychological distress for each pattern 45](#_Toc217142698)

[Table S3. The β diversity pairwise comparison 46](#_Toc217142699)

[Table S4. Regression results for maternal prenatal co-exposure patterns and α diversity and infant neurodevelopment, pattern 2 was set as the reference 47](#_Toc217142700)

[Table S5. Regression results for maternal prenatal co-exposure patterns and α diversity and infant neurodevelopment, pattern 3 was set as the reference 49](#_Toc217142701)

[Table S6. Regression results for the meconium microbiota identified by MaAsLin analysis and infant neurodevelopment, pattern 2 was set as the reference 51](#_Toc217142702)

[Table S7. Regression results for the meconium microbiota identified by MaAsLin analysis and infant neurodevelopment, pattern 3 was set as the reference 52](#_Toc217142703)

[Table S8. MaAsLin differential meconium microbiota screening, pattern 2 was set as the reference 53](#_Toc217142704)

[Table S9. MaAsLin differential meconium microbiota screening, pattern 3 was set as the reference 56](#_Toc217142705)

[Table S10. Power analysis 58](#_Toc217142706)

[Figure S1. SOM results 60](#_Toc217142707)

[Figure S2. Parameters for selecting the number of clusters 61](#_Toc217142708)

[Figure S3. The inter-pattern comparison of maternal prenatal air pollution and psychological distress variables among the three patterns 62](#_Toc217142709)

[Figure S4. Nightingale rose diagram for the visualization of maternal prenatal co-exposure patterns 63](#_Toc217142710)

[Figure S5. Sensitivity analysis 64](#_Toc217142711)

[Figure S6. Additional analysis 66](#_Toc217142712)

[Figure S7. The mediating role of meconium microbiota in maternal prenatal co-exposure patterns and the infant neurodevelopment, pattern 3 was set as the reference 68](#_Toc217142713)

[Figure S8. The importance ranking of 5 air pollution indicators and 3 psychological distress indicators for the meconium microbiota and infant neurodevelopment 69](#_Toc217142714)

[Figure S9. The interaction between maternal prenatal air pollution and psychological distress principal components on meconium microbiota and infant neurodevelopment 70](#_Toc217142715)

[Figure S10. Joint effect of the maternal prenatal co-exposure on infant neurodevelopment total scores at three time points and the significant meconium microbiota in the mediating analysis 71](#_Toc217142716)

Table S1. Quality evaluation of map sizes for each category

| MapSize | QE | TE | DME |
| --- | --- | --- | --- |
| 8 × 10 | 0.243 | 0.476 | 0.005 |
| 9 × 10 | 0.237 | 0.508 | 0.009 |
| 10 × 10 | 0.233 | 0.566 | 0.006 |

QE: quantization error of the map; TE: topographic error; DME: distribution matching error. The smaller the three indices, the higher the quality of the map.

Table S2. The standardized values of maternal prenatal air pollution and psychological distress for each pattern

| Pattern | PM_2.5_ | PM_10_ | CO | NO_2_ | SO_2_ | GAD | EPDS | ISI |
| --- | --- | --- | --- | --- | --- | --- | --- | --- |
| 1 | 0.586 | 0.632 | 0.596 | 0.715 | 0.818 | 0.626 | 0.708 | 0.579 |
| 2 | 0.231 | 0.334 | 0.348 | 0.47 | 0.605 | 0.514 | 0.580 | 0.510 |
| 3 | 0.541 | 0.583 | 0.578 | 0.704 | 0.795 | 0.233 | 0.334 | 0.367 |

Table S3. The β diversity pairwise comparison

|  | *F* | *R^2^* | *p* | *p_Bonf_* |
| --- | --- | --- | --- | --- |
| Phylum level |  |  |  |  |
| Pattern 1 vs Pattern 2 | 9.018 | 0.039 | **0.001** | **0.003** |
| Pattern 1 vs Pattern 3 | 29.770 | 0.137 | **0.001** | **0.003** |
| Pattern 2 vs Pattern 3 | 5.696 | 0.028 | **0.013** | **0.039** |
| Genus level |  |  |  |  |
| Pattern 1 vs Pattern 2 | 19.528 | 0.080 | **0.001** | **0.003** |
| Pattern 1 vs Pattern 3 | 13.004 | 0.065 | **0.001** | **0.003** |
| Pattern 2 vs Pattern 3 | 5.363 | 0.026 | **0.001** | **0.003** |

The *p* values were adjusted for multiple comparisons using the Bonferroni method.

Women in pattern 1 experienced the highest air pollution with the highest psychological distress; women in pattern 2 experienced low pollution with moderate psychological distress; and women in pattern 3 experienced high pollution with low psychological distress.

Table S4. Regression results for maternal prenatal co-exposure patterns and α diversity and infant neurodevelopment, pattern 2 was set as the reference

|  | *N* | Contrast | *B* | *SE* | *p* | *p*_FDR_ |
| --- | --- | --- | --- | --- | --- | --- |
| Chao1 | 309 | Pattern 1 vs Pattern 2 | 0.311 | 0.104 | **0.003** | 0.097 |
| Chao1 | 309 | Pattern 3 vs Pattern 2 | 0.037 | 0.110 | 0.737 | 1.000 |
| Shannon | 309 | Pattern 1 vs Pattern 2 | 0.278 | 0.326 | 0.395 | 0.850 |
| Shannon | 309 | Pattern 3 vs Pattern 2 | 0.356 | 0.344 | 0.301 | 0.850 |
| Simpson | 309 | Pattern 1 vs Pattern 2 | 0.034 | 0.041 | 0.412 | 0.850 |
| Simpson | 309 | Pattern 3 vs Pattern 2 | 0.036 | 0.044 | 0.411 | 0.850 |
| T4 Communication | 283 | Pattern 1 vs Pattern 2 | 0.316 | 0.192 | 0.101 | 0.579 |
| T4 Communication | 283 | Pattern 3 vs Pattern 2 | -0.275 | 0.202 | 0.173 | 0.646 |
| T4 Gross motor ability | 283 | Pattern 1 vs Pattern 2 | -0.044 | 0.201 | 0.826 | 0.956 |
| T4 Gross motor ability | 283 | Pattern 3 vs Pattern 2 | -0.697 | 0.211 | **0.001** | 0.081 |
| T4 Fine motor ability | 278 | Pattern 1 vs Pattern 2 | -0.082 | 0.164 | 0.620 | 0.941 |
| T4 Fine motor ability | 278 | Pattern 3 vs Pattern 2 | -0.454 | 0.173 | **0.009** | 0.202 |
| T4 Problem-solving ability | 283 | Pattern 1 vs Pattern 2 | -0.118 | 0.194 | 0.543 | 0.914 |
| T4 Problem-solving ability | 283 | Pattern 3 vs Pattern 2 | -0.517 | 0.204 | **0.012** | 0.226 |
| T4 Personal and social ability | 282 | Pattern 1 vs Pattern 2 | 0.118 | 0.174 | 0.496 | 0.903 |
| T4 Personal and social ability | 282 | Pattern 3 vs Pattern 2 | -0.355 | 0.183 | 0.054 | 0.497 |
| T4 ASQ total score | 277 | Pattern 1 vs Pattern 2 | 0.073 | 0.161 | 0.650 | 0.941 |
| T4 ASQ total score | 277 | Pattern 3 vs Pattern 2 | -0.357 | 0.170 | **0.037** | 0.390 |
| T5 Communication | 274 | Pattern 1 vs Pattern 2 | 0.166 | 0.169 | 0.328 | 0.830 |
| T5 Communication | 274 | Pattern 3 vs Pattern 2 | 0.201 | 0.185 | 0.279 | 0.813 |
| T5 Gross motor ability | 274 | Pattern 1 vs Pattern 2 | 0.274 | 0.193 | 0.157 | 0.646 |
| T5 Gross motor ability | 274 | Pattern 3 vs Pattern 2 | -0.108 | 0.210 | 0.607 | 0.941 |
| T5 Fine motor ability | 274 | Pattern 1 vs Pattern 2 | 0.294 | 0.193 | 0.128 | 0.621 |
| T5 Fine motor ability | 274 | Pattern 3 vs Pattern 2 | -0.147 | 0.210 | 0.486 | 0.903 |
| T5 Problem-solving ability | 274 | Pattern 1 vs Pattern 2 | 0.245 | 0.194 | 0.206 | 0.691 |
| T5 Problem-solving ability | 274 | Pattern 3 vs Pattern 2 | -0.175 | 0.211 | 0.408 | 0.877 |
| T5 Personal and social ability | 274 | Pattern 1 vs Pattern 2 | 0.603 | 0.177 | **0.001** | 0.081 |
| T5 Personal and social ability | 274 | Pattern 3 vs Pattern 2 | 0.188 | 0.193 | 0.331 | 0.830 |
| T5 ASQ total score | 274 | Pattern 1 vs Pattern 2 | 0.450 | 0.194 | **0.021** | 0.301 |
| T5 ASQ total score | 274 | Pattern 3 vs Pattern 2 | 0.089 | 0.212 | 0.676 | 0.941 |
| T6 Communication | 264 | Pattern 1 vs Pattern 2 | 0.322 | 0.176 | 0.069 | 0.529 |
| T6 Communication | 264 | Pattern 3 vs Pattern 2 | -0.072 | 0.203 | 0.723 | 0.947 |
| T6 Gross motor ability | 265 | Pattern 1 vs Pattern 2 | -0.257 | 0.182 | 0.159 | 0.646 |
| T6 Gross motor ability | 265 | Pattern 3 vs Pattern 2 | -0.331 | 0.210 | 0.117 | 0.621 |
| T6 Fine motor ability | 265 | Pattern 1 vs Pattern 2 | -0.177 | 0.193 | 0.360 | 0.844 |
| T6 Fine motor ability | 265 | Pattern 3 vs Pattern 2 | -0.625 | 0.223 | **0.005** | 0.194 |
| T6 Problem-solving ability | 265 | Pattern 1 vs Pattern 2 | -0.083 | 0.189 | 0.659 | 0.941 |
| T6 Problem-solving ability | 265 | Pattern 3 vs Pattern 2 | -0.129 | 0.218 | 0.555 | 0.930 |
| T6 Personal and social ability | 265 | Pattern 1 vs Pattern 2 | 0.063 | 0.187 | 0.738 | 0.947 |
| T6 Personal and social ability | 265 | Pattern 3 vs Pattern 2 | -0.251 | 0.216 | 0.247 | 0.791 |
| T6 ASQ total score | 264 | Pattern 1 vs Pattern 2 | -0.032 | 0.169 | 0.849 | 0.956 |
| T6 ASQ total score | 264 | Pattern 3 vs Pattern 2 | -0.339 | 0.195 | 0.083 | 0.564 |

T4, 1 months postpartum; T5, 3 months postpartum; T6, 6 months postpartum.

In the infant neurodevelopment model, maternal age, maternal education level, average monthly household income, pre-pregnancy body mass index, weight gain during pregnancy, complications during pregnancy, smoking during pregnancy, drinking during pregnancy, infant sex, delivery mode, infant gestational age at birth, and infant birth weight were adjusted.

In the meconium microbiota α diversity model, we adjusted for additional variables, including group B streptococci, vaginitis, frequency of intake of vegetables and fruits, frequency of intake of high-quality protein, and fetal feces collection time.

Table S5. Regression results for maternal prenatal co-exposure patterns and α diversity and infant neurodevelopment, pattern 3 was set as the reference

|  | *N* | Contrast | *B* | *SE* | *p* | *p*_FDR_ |
| --- | --- | --- | --- | --- | --- | --- |
| Chao1 | 309 | Pattern 1 vs Pattern 3 | 0.274 | 0.113 | **0.016** | 0.498 |
| Chao1 | 309 | Pattern 2 vs Pattern 3 | -0.037 | 0.110 | 0.737 | 1.000 |
| Shannon | 309 | Pattern 1 vs Pattern 3 | -0.079 | 0.354 | 0.825 | 1.000 |
| Shannon | 309 | Pattern 2 vs Pattern 3 | -0.356 | 0.344 | 0.301 | 0.896 |
| Simpson | 309 | Pattern 1 vs Pattern 3 | -0.002 | 0.045 | 0.967 | 1.000 |
| Simpson | 309 | Pattern 2 vs Pattern 3 | -0.036 | 0.044 | 0.411 | 0.904 |
| T4 Communication | 283 | Pattern 1 vs Pattern 3 | 0.592 | 0.207 | **0.004** | 0.162 |
| T4 Communication | 283 | Pattern 2 vs Pattern 3 | 0.275 | 0.202 | 0.173 | 0.586 |
| T4 Gross motor ability | 283 | Pattern 1 vs Pattern 3 | 0.653 | 0.216 | **0.003** | 0.149 |
| T4 Gross motor ability | 283 | Pattern 2 vs Pattern 3 | 0.697 | 0.211 | **0.001** | 0.122 |
| T4 Fine motor ability | 278 | Pattern 1 vs Pattern 3 | 0.372 | 0.176 | **0.036** | 0.352 |
| T4 Fine motor ability | 278 | Pattern 2 vs Pattern 3 | 0.454 | 0.173 | **0.009** | 0.188 |
| T4 Problem-solving ability | 283 | Pattern 1 vs Pattern 3 | 0.398 | 0.209 | 0.057 | 0.431 |
| T4 Problem-solving ability | 283 | Pattern 2 vs Pattern 3 | 0.517 | 0.204 | **0.012** | 0.220 |
| T4 Personal and social ability | 282 | Pattern 1 vs Pattern 3 | 0.474 | 0.188 | **0.012** | 0.220 |
| T4 Personal and social ability | 282 | Pattern 2 vs Pattern 3 | 0.355 | 0.183 | 0.054 | 0.428 |
| T4 ASQ total score | 277 | Pattern 1 vs Pattern 3 | 0.431 | 0.174 | **0.014** | 0.234 |
| T4 ASQ total score | 277 | Pattern 2 vs Pattern 3 | 0.357 | 0.170 | **0.037** | 0.352 |
| T5 Communication | 274 | Pattern 1 vs Pattern 3 | -0.035 | 0.190 | 0.855 | 0.961 |
| T5 Communication | 274 | Pattern 2 vs Pattern 3 | -0.201 | 0.185 | 0.279 | 0.759 |
| T5 Gross motor ability | 274 | Pattern 1 vs Pattern 3 | 0.382 | 0.216 | 0.078 | 0.467 |
| T5 Gross motor ability | 274 | Pattern 2 vs Pattern 3 | 0.108 | 0.210 | 0.607 | 0.934 |
| T5 Fine motor ability | 274 | Pattern 1 vs Pattern 3 | 0.441 | 0.216 | **0.042** | 0.378 |
| T5 Fine motor ability | 274 | Pattern 2 vs Pattern 3 | 0.147 | 0.210 | 0.486 | 0.887 |
| T5 Problem-solving ability | 274 | Pattern 1 vs Pattern 3 | 0.421 | 0.217 | 0.053 | 0.428 |
| T5 Problem-solving ability | 274 | Pattern 2 vs Pattern 3 | 0.175 | 0.211 | 0.408 | 0.839 |
| T5 Personal and social ability | 274 | Pattern 1 vs Pattern 3 | 0.415 | 0.198 | **0.037** | 0.352 |
| T5 Personal and social ability | 274 | Pattern 2 vs Pattern 3 | -0.188 | 0.193 | 0.331 | 0.789 |
| T5 ASQ total score | 274 | Pattern 1 vs Pattern 3 | 0.362 | 0.217 | 0.097 | 0.475 |
| T5 ASQ total score | 274 | Pattern 2 vs Pattern 3 | -0.089 | 0.212 | 0.676 | 0.943 |
| T6 Communication | 264 | Pattern 1 vs Pattern 3 | 0.394 | 0.210 | 0.062 | 0.431 |
| T6 Communication | 264 | Pattern 2 vs Pattern 3 | 0.072 | 0.203 | 0.723 | 0.947 |
| T6 Gross motor ability | 265 | Pattern 1 vs Pattern 3 | 0.074 | 0.218 | 0.736 | 0.947 |
| T6 Gross motor ability | 265 | Pattern 2 vs Pattern 3 | 0.331 | 0.210 | 0.117 | 0.535 |
| T6 Fine motor ability | 265 | Pattern 1 vs Pattern 3 | 0.448 | 0.231 | 0.053 | 0.428 |
| T6 Fine motor ability | 265 | Pattern 2 vs Pattern 3 | 0.625 | 0.223 | **0.005** | 0.173 |
| T6 Problem-solving ability | 265 | Pattern 1 vs Pattern 3 | 0.045 | 0.226 | 0.841 | 0.958 |
| T6 Problem-solving ability | 265 | Pattern 2 vs Pattern 3 | 0.129 | 0.218 | 0.555 | 0.914 |
| T6 Personal and social ability | 265 | Pattern 1 vs Pattern 3 | 0.313 | 0.224 | 0.163 | 0.586 |
| T6 Personal and social ability | 265 | Pattern 2 vs Pattern 3 | 0.251 | 0.216 | 0.247 | 0.734 |
| T6 ASQ total score | 264 | Pattern 1 vs Pattern 3 | 0.307 | 0.202 | 0.129 | 0.540 |
| T6 ASQ total score | 264 | Pattern 2 vs Pattern 3 | 0.339 | 0.195 | 0.083 | 0.475 |

T4, 1 months postpartum; T5, 3 months postpartum; T6, 6 months postpartum.

In the infant neurodevelopment model, maternal age, maternal education level, average monthly household income, pre-pregnancy body mass index, weight gain during pregnancy, complications during pregnancy, smoking during pregnancy, drinking during pregnancy, infant sex, delivery mode, infant gestational age at birth, and infant birth weight were adjusted.

In the meconium microbiota α diversity model, we adjusted for additional variables including group B streptococci, vaginitis, frequency of intake of vegetables and fruits, frequency of intake of high-quality protein, and fetal feces collection time.

Table S6. Regression results for the meconium microbiota identified by MaAsLin analysis and infant neurodevelopment, pattern 2 was set as the reference

| Meconium microbiota | Infant neurodevelopment | *N* | *B* | *SE* | *p* | *p_FDR_* |
| --- | --- | --- | --- | --- | --- | --- |
| g_*unclassified_Lachnospiraceae* | T5 Personal and social ability | 274 | -56.247 | 14.785 | **0.000** | **0.018** |
| g_*Ruminococcus* | T5 Fine motor ability | 274 | -108.085 | 22.345 | **0.000** | **0.000** |
| g_*Ruminococcus* | T5 Personal and social ability | 274 | -104.019 | 20.707 | **0.000** | **0.000** |
| g_*Ruminococcus* | T5 ASQ total score | 274 | -104.533 | 22.393 | **0.000** | **0.001** |
| g_*Ruminococcus* | T6 ASQ total score | 264 | -40.055 | 10.876 | **0.000** | **0.022** |

T5, 3 months postpartum; T6, 6 months postpartum.

Using 396 regression models, we evaluated the associations between 22 meconium microbiotas identified by MaAsLin and 18 infant neurodevelopmental outcomes (ASQ-3 total scores and five domains at three time points). Only FDR-corrected significant results (*p_FDR_* < 0.05) are presented.

The models were adjusted for maternal age, maternal education level, average monthly household income, pre-pregnancy body mass index, weight gain during pregnancy, complications during pregnancy, smoking during pregnancy, drinking during pregnancy, infant sex, delivery mode, infant gestational age at birth, infant birth weight, group B streptococci, vaginitis, frequency of intake of vegetables and fruits, frequency of intake of high-quality protein, and fetal feces collection time.

Table S7. Regression results for the meconium microbiota identified by MaAsLin analysis and infant neurodevelopment, pattern 3 was set as the reference

| Meconium microbiota | Infant neurodevelopment | *N* | *B* | *SE* | *p* | *p_FDR_* |
| --- | --- | --- | --- | --- | --- | --- |
| g_*unclassified_Lachnospiraceae* | T5 Personal and social ability | 274 | -56.247 | 14.785 | **0.000** | **0.011** |
| g_*Ruminococcus* | T5 Fine motor ability | 274 | -108.085 | 22.345 | **0.000** | **0.000** |
| g_*Ruminococcus* | T5 Problem-solving ability | 274 | -75.707 | 22.698 | **0.001** | **0.041** |
| g_*Ruminococcus* | T5 Personal and social ability | 274 | -104.019 | 20.707 | **0.000** | **0.000** |
| g_*Ruminococcus* | T5 ASQ total score | 274 | -104.533 | 22.393 | **0.000** | **0.000** |
| g_*Ruminococcus* | T6 ASQ total score | 264 | -40.055 | 10.876 | **0.000** | **0.014** |

T5, 3 months postpartum; T6, 6 months postpartum.

Using 252 regression models, we evaluated the associations between 14 meconium microbiotas identified by MaAsLin and 18 infant neurodevelopmental outcomes (ASQ-3 total scores and five domains at three time points). Only FDR-corrected significant results (*p_FDR_* < 0.05) are presented.

The models were adjusted for maternal age, maternal education level, average monthly household income, pre-pregnancy body mass index, weight gain during pregnancy, complications during pregnancy, smoking during pregnancy, drinking during pregnancy, infant sex, delivery mode, infant gestational age at birth, infant birth weight, group B streptococci, vaginitis, frequency of intake of vegetables and fruits, frequency of intake of high-quality protein, and fetal feces collection time.

Table S8. MaAsLin differential meconium microbiota screening, pattern 2 was set as the reference

| Pattern | Feature | *B* | *SE* | *N* | Non zero N | *p* | *p*_FDR_ |
| --- | --- | --- | --- | --- | --- | --- | --- |
| Pattern 1 | d__Bacteria\|p__Bacteroidetes | -2.393 | 0.357 | 309 | 308 | **0.000** | **0.000** |
| Pattern 3 | d__Bacteria\|p__Bacteroidetes | -1.558 | 0.376 | 309 | 308 | **0.000** | **0.002** |
| Pattern 1 | d__Bacteria\|p__Bacteroidetes\|c__Bacteroidia\|o__Bacteroidales\|f__Porphyromonadaceae\|g__Parabacteroides | -1.779 | 0.429 | 309 | 260 | **0.000** | **0.001** |
| Pattern 3 | d__Bacteria\|p__Bacteroidetes\|c__Bacteroidia\|o__Bacteroidales\|f__Porphyromonadaceae\|g__Parabacteroides | -1.689 | 0.453 | 309 | 260 | **0.000** | **0.004** |
| Pattern 1 | d__Bacteria\|p__Bacteroidetes\|c__Bacteroidia\|o__Bacteroidales\|f__Prevotellaceae\|g__Prevotella | -2.787 | 0.525 | 309 | 269 | **0.000** | **0.000** |
| Pattern 3 | d__Bacteria\|p__Bacteroidetes\|c__Bacteroidia\|o__Bacteroidales\|f__Prevotellaceae\|g__Prevotella | -1.911 | 0.553 | 309 | 269 | **0.001** | **0.009** |
| Pattern 1 | d__Bacteria\|p__Bacteroidetes\|c__Bacteroidia\|o__Bacteroidales\|f__S24-7\|g__unidentified_S24-7 | -3.170 | 0.529 | 309 | 295 | **0.000** | **0.000** |
| Pattern 3 | d__Bacteria\|p__Bacteroidetes\|c__Bacteroidia\|o__Bacteroidales\|f__S24-7\|g__unidentified_S24-7 | -2.453 | 0.558 | 309 | 295 | **0.000** | **0.000** |
| Pattern 1 | d__Bacteria\|p__Bacteroidetes\|c__Bacteroidia\|o__Bacteroidales\|f__unidentified_Bacteroidales\|g__unidentified_Bacteroidales | -3.779 | 0.508 | 309 | 272 | **0.000** | **0.000** |
| Pattern 3 | d__Bacteria\|p__Bacteroidetes\|c__Bacteroidia\|o__Bacteroidales\|f__unidentified_Bacteroidales\|g__unidentified_Bacteroidales | -3.118 | 0.535 | 309 | 272 | **0.000** | **0.000** |
| Pattern 1 | d__Bacteria\|p__Firmicutes | -1.567 | 0.330 | 309 | 309 | **0.000** | **0.000** |
| Pattern 1 | d__Bacteria\|p__Firmicutes\|c__Bacilli\|o__Lactobacillales\|f__Lactobacillaceae\|g__Lactobacillus | -1.994 | 0.361 | 309 | 308 | **0.000** | **0.000** |
| Pattern 3 | d__Bacteria\|p__Firmicutes\|c__Bacilli\|o__Lactobacillales\|f__Lactobacillaceae\|g__Lactobacillus | -1.694 | 0.381 | 309 | 308 | **0.000** | **0.000** |
| Pattern 1 | d__Bacteria\|p__Firmicutes\|c__Bacilli\|o__Lactobacillales\|f__Streptococcaceae\|g__Streptococcus | -2.444 | 0.445 | 309 | 289 | **0.000** | **0.000** |
| Pattern 1 | d__Bacteria\|p__Firmicutes\|c__Clostridia\|o__Clostridiales\|f__Lachnospiraceae\|g__Coprococcus | -2.994 | 0.446 | 309 | 243 | **0.000** | **0.000** |
| Pattern 3 | d__Bacteria\|p__Firmicutes\|c__Clostridia\|o__Clostridiales\|f__Lachnospiraceae\|g__Coprococcus | -2.659 | 0.470 | 309 | 243 | **0.000** | **0.000** |
| Pattern 1 | d__Bacteria\|p__Firmicutes\|c__Clostridia\|o__Clostridiales\|f__Lachnospiraceae\|g__unclassified_Lachnospiraceae | -2.496 | 0.539 | 309 | 263 | **0.000** | **0.000** |
| Pattern 3 | d__Bacteria\|p__Firmicutes\|c__Clostridia\|o__Clostridiales\|f__Lachnospiraceae\|g__unclassified_Lachnospiraceae | -2.105 | 0.568 | 309 | 263 | **0.000** | **0.005** |
| Pattern 1 | d__Bacteria\|p__Firmicutes\|c__Clostridia\|o__Clostridiales\|f__Lachnospiraceae\|g__unidentified_Lachnospiraceae | -2.752 | 0.486 | 309 | 284 | **0.000** | **0.000** |
| Pattern 3 | d__Bacteria\|p__Firmicutes\|c__Clostridia\|o__Clostridiales\|f__Lachnospiraceae\|g__unidentified_Lachnospiraceae | -1.812 | 0.512 | 309 | 284 | **0.000** | **0.007** |
| Pattern 1 | d__Bacteria\|p__Firmicutes\|c__Clostridia\|o__Clostridiales\|f__Ruminococcaceae\|g__Oscillospira | -2.921 | 0.480 | 309 | 277 | **0.000** | **0.000** |
| Pattern 3 | d__Bacteria\|p__Firmicutes\|c__Clostridia\|o__Clostridiales\|f__Ruminococcaceae\|g__Oscillospira | -2.636 | 0.506 | 309 | 277 | **0.000** | **0.000** |
| Pattern 1 | d__Bacteria\|p__Firmicutes\|c__Clostridia\|o__Clostridiales\|f__Ruminococcaceae\|g__Ruminococcus | -2.476 | 0.459 | 309 | 275 | **0.000** | **0.000** |
| Pattern 3 | d__Bacteria\|p__Firmicutes\|c__Clostridia\|o__Clostridiales\|f__Ruminococcaceae\|g__Ruminococcus | -2.180 | 0.484 | 309 | 275 | **0.000** | **0.000** |
| Pattern 1 | d__Bacteria\|p__Firmicutes\|c__Clostridia\|o__Clostridiales\|f__Ruminococcaceae\|g__unclassified_Ruminococcaceae | -2.028 | 0.459 | 309 | 264 | **0.000** | **0.000** |
| Pattern 3 | d__Bacteria\|p__Firmicutes\|c__Clostridia\|o__Clostridiales\|f__Ruminococcaceae\|g__unclassified_Ruminococcaceae | -1.908 | 0.484 | 309 | 264 | **0.000** | **0.002** |
| Pattern 1 | d__Bacteria\|p__Firmicutes\|c__Clostridia\|o__Clostridiales\|f__Ruminococcaceae\|g__unidentified_Ruminococcaceae | -2.381 | 0.506 | 309 | 284 | **0.000** | **0.000** |
| Pattern 3 | d__Bacteria\|p__Firmicutes\|c__Clostridia\|o__Clostridiales\|f__Ruminococcaceae\|g__unidentified_Ruminococcaceae | -1.899 | 0.533 | 309 | 284 | **0.000** | **0.007** |
| Pattern 1 | d__Bacteria\|p__Firmicutes\|c__Clostridia\|o__Clostridiales\|f__unidentified_Clostridiales\|g__unidentified_Clostridiales | -2.258 | 0.464 | 309 | 274 | **0.000** | **0.000** |
| Pattern 1 | d__Bacteria\|p__Firmicutes\|c__Clostridia\|o__Clostridiales\|f__unclassified_Clostridiales\|g__unclassified_Clostridiales | -2.813 | 0.478 | 309 | 284 | **0.000** | **0.000** |
| Pattern 3 | d__Bacteria\|p__Firmicutes\|c__Clostridia\|o__Clostridiales\|f__unclassified_Clostridiales\|g__unclassified_Clostridiales | -1.977 | 0.504 | 309 | 284 | **0.000** | **0.002** |
| Pattern 1 | d__Bacteria\|p__Proteobacteria\|c__Betaproteobacteria\|o__Burkholderiales\|f__Comamonadaceae\|g__Acidovorax | -1.676 | 0.364 | 309 | 283 | **0.000** | **0.000** |
| Pattern 3 | d__Bacteria\|p__Proteobacteria\|c__Betaproteobacteria\|o__Burkholderiales\|f__Comamonadaceae\|g__Acidovorax | -1.686 | 0.383 | 309 | 283 | **0.000** | **0.000** |
| Pattern 1 | d__Bacteria\|p__Proteobacteria\|c__Betaproteobacteria\|o__Burkholderiales\|f__Comamonadaceae\|g__Aquabacterium | -1.756 | 0.511 | 309 | 277 | **0.001** | **0.009** |
| Pattern 3 | d__Bacteria\|p__Proteobacteria\|c__Betaproteobacteria\|o__Burkholderiales\|f__Comamonadaceae\|g__Aquabacterium | -1.868 | 0.539 | 309 | 277 | **0.001** | **0.008** |
| Pattern 3 | d__Bacteria\|p__Proteobacteria\|c__Betaproteobacteria\|o__Burkholderiales\|f__Comamonadaceae\|g__Pelomonas | 2.475 | 0.610 | 309 | 269 | **0.000** | **0.001** |
| Pattern 1 | d__Bacteria\|p__Proteobacteria\|c__Betaproteobacteria\|o__Burkholderiales\|f__Comamonadaceae\|g__Rubrivivax | -3.148 | 0.479 | 309 | 284 | **0.000** | **0.000** |
| Pattern 3 | d__Bacteria\|p__Proteobacteria\|c__Betaproteobacteria\|o__Burkholderiales\|f__Comamonadaceae\|g__Rubrivivax | -3.223 | 0.505 | 309 | 284 | **0.000** | **0.000** |
| Pattern 1 | d__Bacteria\|p__Proteobacteria\|c__Gammaproteobacteria\|o__Enterobacteriales\|f__Enterobacteriaceae\|g__unclassified_Enterobacteriaceae | 1.428 | 0.398 | 309 | 301 | **0.000** | **0.006** |

Adjusted for maternal age, maternal education level, average monthly household income, pre-pregnancy body mass index, weight gain during pregnancy, complications during pregnancy, smoking during pregnancy, drinking during pregnancy, infant sex, delivery mode, infant gestational age at birth, infant birth weight, group B streptococci, vaginitis, delivery mode, frequency of intake of vegetables and fruits, frequency of intake of high-quality protein, and fetal feces collection time.

Table S9. MaAsLin differential meconium microbiota screening, pattern 3 was set as the reference

| Pattern | Feature | *B* | *SE* | *N* | Non zero N | *p* | *p*_FDR_ |
| --- | --- | --- | --- | --- | --- | --- | --- |
| Pattern 2 | d__Bacteria\|p__Bacteroidetes | 1.558 | 0.376 | 309 | 308 | **0.000** | **0.005** |
| Pattern 2 | d__Bacteria\|p__Bacteroidetes\|c__Bacteroidia\|o__Bacteroidales\|f__Porphyromonadaceae\|g__Parabacteroides | 1.689 | 0.453 | 309 | 260 | **0.000** | **0.009** |
| Pattern 2 | d__Bacteria\|p__Bacteroidetes\|c__Bacteroidia\|o__Bacteroidales\|f__S24-7\|g__unidentified_S24-7 | 2.453 | 0.558 | 309 | 295 | **0.000** | **0.001** |
| Pattern 2 | d__Bacteria\|p__Bacteroidetes\|c__Bacteroidia\|o__Bacteroidales\|f__unidentified_Bacteroidales\|g__unidentified_Bacteroidales | 3.118 | 0.535 | 309 | 272 | **0.000** | **0.000** |
| Pattern 2 | d__Bacteria\|p__Firmicutes\|c__Bacilli\|o__Lactobacillales\|f__Lactobacillaceae\|g__Lactobacillus | 1.694 | 0.381 | 309 | 308 | **0.000** | **0.001** |
| Pattern 2 | d__Bacteria\|p__Firmicutes\|c__Clostridia\|o__Clostridiales\|f__Lachnospiraceae\|g__Coprococcus | 2.659 | 0.470 | 309 | 243 | **0.000** | **0.000** |
| Pattern 2 | d__Bacteria\|p__Firmicutes\|c__Clostridia\|o__Clostridiales\|f__Lachnospiraceae\|g__unclassified_Lachnospiraceae | 2.105 | 0.568 | 309 | 263 | **0.000** | **0.010** |
| Pattern 2 | d__Bacteria\|p__Firmicutes\|c__Clostridia\|o__Clostridiales\|f__Ruminococcaceae\|g__Oscillospira | 2.636 | 0.506 | 309 | 277 | **0.000** | **0.000** |
| Pattern 2 | d__Bacteria\|p__Firmicutes\|c__Clostridia\|o__Clostridiales\|f__Ruminococcaceae\|g__Ruminococcus | 2.180 | 0.484 | 309 | 275 | **0.000** | **0.001** |
| Pattern 2 | d__Bacteria\|p__Firmicutes\|c__Clostridia\|o__Clostridiales\|f__Ruminococcaceae\|g__unclassified_Ruminococcaceae | 1.908 | 0.484 | 309 | 264 | **0.000** | **0.005** |
| Pattern 2 | d__Bacteria\|p__Firmicutes\|c__Clostridia\|o__Clostridiales\|f__unclassified_Clostridiales\|g__unclassified_Clostridiales | 1.977 | 0.504 | 309 | 284 | **0.000** | **0.005** |
| Pattern 2 | d__Bacteria\|p__Proteobacteria\|c__Betaproteobacteria\|o__Burkholderiales\|f__Comamonadaceae\|g__Acidovorax | 1.686 | 0.383 | 309 | 283 | **0.000** | **0.001** |
| Pattern 2 | d__Bacteria\|p__Proteobacteria\|c__Betaproteobacteria\|o__Burkholderiales\|f__Comamonadaceae\|g__Pelomonas | -2.475 | 0.610 | 309 | 269 | **0.000** | **0.004** |
| Pattern 2 | d__Bacteria\|p__Proteobacteria\|c__Betaproteobacteria\|o__Burkholderiales\|f__Comamonadaceae\|g__Rubrivivax | 3.223 | 0.505 | 309 | 284 | **0.000** | **0.000** |

Adjusted for maternal age, maternal education level, average monthly household income, pre-pregnancy body mass index, weight gain during pregnancy, complications during pregnancy, smoking during pregnancy, drinking during pregnancy, infant sex, delivery mode, infant gestational age at birth, infant birth weight, group B streptococci, vaginitis, delivery mode, frequency of intake of vegetables and fruits, frequency of intake of high-quality protein, and fetal feces collection time.

Table S10. Power analysis

|  | *N* | std_a | std_b | std_c' | Power_a*b |
| --- | --- | --- | --- | --- | --- |
| Analysis1. Conducting mediation analysis using the exposure patterns formed by the average |  |  |  |  |  |
| Pattern 1_vs Pattern 2=>g_*Ruminococcus*=>T5 ASQ total score | 274 | -0.308 | -0.256 | 0.080 | 99% |
| Pattern 3_vs Pattern 2=>g_*Ruminococcus*=>T5 ASQ total score | 274 | -0.273 | -0.256 | -0.010 | 98% |
| Pattern 1_vs Pattern 2=>g_*Ruminococcus*=>T5 Fine motor ability | 274 | -0.308 | -0.291 | 0.018 | 100% |
| Pattern 3_vs Pattern 2=>g_*Ruminococcus*=>T5 Fine motor ability | 274 | -0.273 | -0.291 | -0.100 | 99% |
| Pattern 1_vs Pattern 2=>g_*Ruminococcus*=>T5 Problem-solving ability | 274 | -0.308 | -0.205 | 0.017 | 91% |
| Pattern 3_vs Pattern 2=>g_*Ruminococcus*=>T5 Problem-solving ability | 274 | -0.273 | -0.205 | -0.085 | 91% |
| Pattern 1_vs Pattern 2=>g_*Ruminococcus*=>T5 Personal-social ability | 274 | -0.308 | -0.260 | 0.160 | 99% |
| Pattern 3_vs Pattern 2=>g_*Ruminococcus*=>T5 Personal-social ability | 274 | -0.273 | -0.260 | 0.022 | 99% |
| Analysis2. Conducting mediation analysis using the exposure patterns formed by the average while reducing the number of covariates |  |  |  |  |  |
| Pattern 1_vs Pattern 2=>g_*Ruminococcus*=>T5 ASQ total score | 274 | -0.311 | -0.259 | 0.085 | 99% |
| Pattern 3_vs Pattern 2=>g_*Ruminococcus*=>T5 ASQ total score | 274 | -0.268 | -0.259 | -0.037 | 98% |
| Pattern 1_vs Pattern 2=>g_*Ruminococcus*=>T5 Fine motor ability | 274 | -0.311 | -0.291 | 0.034 | 100% |
| Pattern 3_vs Pattern 2=>g_*Ruminococcus*=>T5 Fine motor ability | 274 | -0.268 | -0.291 | -0.113 | 99% |
| Pattern 1_vs Pattern 2=>g_*Ruminococcus*=>T5 Problem-solving ability | 274 | -0.311 | -0.203 | 0.036 | 90% |
| Pattern 3_vs Pattern 2=>g_*Ruminococcus*=>T5 Problem-solving ability | 274 | -0.268 | -0.203 | -0.094 | 90% |
| Pattern 1_vs Pattern 2=>g_*Ruminococcus*=>T5 Personal-social ability | 274 | -0.311 | -0.259 | 0.153 | 99% |
| Pattern 3_vs Pattern 2=>g_*Ruminococcus*=>T5 Personal-social ability | 274 | -0.268 | -0.259 | 0.007 | 98% |
| Analysis3. Participants with pregnancy-related complications were excluded for the mediation analysis |  |  |  |  |  |
| Pattern 1_vs Pattern 3=>g_*Ruminococcus*=>T5 ASQ total score | 208 | -0.353 | -0.238 | 0.123 | 92% |
| Pattern 2_vs Pattern 3=>g_*Ruminococcus*=>T5 ASQ total score | 208 | -0.295 | -0.238 | -0.008 | 91% |
| Pattern 1_vs Pattern 3=>g_*Ruminococcus*=>T5 Fine motor ability | 208 | -0.353 | -0.273 | 0.114 | 97% |
| Pattern 2_vs Pattern 3=>g_*Ruminococcus*=>T5 Fine motor ability | 208 | -0.295 | -0.273 | -0.046 | 96% |
| Pattern 1_vs Pattern 3=>g_*Ruminococcus*=>T5 Personal-social ability | 208 | -0.353 | -0.245 | 0.151 | 93% |
| Pattern 2_vs Pattern 3=>g_*Ruminococcus*=>T5 Personal-social ability | 208 | -0.295 | -0.245 | 0.019 | 93% |
| Analysis4. Participants with pregnancy-related complications were excluded for the mediation analysis, and the number of covariates was reduced. |  |  |  |  |  |
| Pattern 1_vs Pattern 3=>g_*Ruminococcus*=>T5 ASQ total score | 208 | -0.358 | -0.238 | 0.148 | 92% |
| Pattern 2_vs Pattern 3=>g_*Ruminococcus*=>T5 ASQ total score | 208 | -0.302 | -0.238 | -0.008 | 91% |
| Pattern 1_vs Pattern 3=>g_*Ruminococcus*=>T5 Fine motor ability | 208 | -0.358 | -0.274 | 0.122 | 97% |
| Pattern 2_vs Pattern 3=>g_*Ruminococcus*=>T5 Fine motor ability | 208 | -0.302 | -0.274 | -0.056 | 97% |
| Pattern 1_vs Pattern 3=>g_*Ruminococcus*=>T5 Personal-social ability | 208 | -0.358 | -0.240 | 0.165 | 93% |
| Pattern 2_vs Pattern 3=>g_*Ruminococcus*=>T5 Personal-social ability | 208 | -0.302 | -0.240 | 0.030 | 92% |
| Analysis5. Conducting mediation analysis using the exposure patterns formed by standard deviation |  |  |  |  |  |
| Pattern 1_vs Pattern 2=>g_*Ruminococcus*=>T5 ASQ total score | 274 | -0.314 | -0.233 | 0.118 | 97% |
| Pattern 1_vs Pattern 2=>g_*Ruminococcus*=>T6 ASQ total score | 264 | -0.241 | -0.250 | -0.102 | 96% |
| Pattern 1_vs Pattern 2=>g_*Ruminococcus*=>T5 Fine motor ability | 274 | -0.314 | -0.254 | 0.084 | 99% |
| Pattern 1_vs Pattern 2=>g_*Ruminococcus*=>T5 Personal-social ability | 274 | -0.314 | -0.249 | 0.137 | 98% |
| Analysis6. Conducting mediation analysis using the exposure patterns formed by standard deviation while reducing the number of covariates |  |  |  |  |  |
| Pattern 1_vs Pattern 2=>g_*Ruminococcus*=>T5 ASQ total score | 274 | -0.314 | -0.230 | 0.128 | 96% |
| Pattern 1_vs Pattern 2=>g_*Ruminococcus*=>T6 ASQ total score | 264 | -0.244 | -0.242 | -0.102 | 96% |
| Pattern 1_vs Pattern 2=>g_*Ruminococcus*=>T5 Fine motor ability | 274 | -0.314 | -0.252 | 0.094 | 98% |
| Pattern 1_vs Pattern 2=>g_*Ruminococcus*=>T5 Personal-social ability | 274 | -0.314 | -0.245 | 0.142 | 98% |
| Analysis7. Conducting mediation analysis using the exposure patterns formed by three time points |  |  |  |  |  |
| Pattern 2_vs Pattern 1=>g_*Ruminococcus*=>T6 ASQ total score | 264 | -0.391 | -0.231 | -0.032 | 94% |
| Pattern 3_vs Pattern 1=>g_*Ruminococcus*=>T6 ASQ total score | 264 | -0.368 | -0.231 | 0.004 | 95% |
| Pattern 2_vs Pattern 1=>g_*Ruminococcus*=>T5 Fine motor ability | 274 | -0.550 | -0.213 | 0.220 | 88% |
| Pattern 3_vs Pattern 1=>g_*Ruminococcus*=>T5 Fine motor ability | 274 | -0.523 | -0.213 | 0.078 | 87% |
| Pattern 2_vs Pattern 1=>g_*Ruminococcus*=>T5 Personal-social ability | 274 | -0.550 | -0.220 | 0.144 | 89% |
| Pattern 3_vs Pattern 1=>g_*Ruminococcus*=>T5 Personal-social ability | 274 | -0.523 | -0.220 | 0.209 | 91% |
| Analysis8. Conducting mediation analysis using the exposure patterns formed by three time points while reducing the number of covariates |  |  |  |  |  |
| Pattern 2_vs Pattern 1=>g_*Ruminococcus*=>T6 ASQ total score | 264 | -0.388 | -0.221 | -0.024 | 92% |
| Pattern 3_vs Pattern 1=>g_*Ruminococcus*=>T6 ASQ total score | 264 | -0.362 | -0.221 | 0.003 | 93% |
| Pattern 2_vs Pattern 1=>g_*Ruminococcus*=>T5 Fine motor ability | 274 | -0.547 | -0.209 | 0.225 | 87% |
| Pattern 3_vs Pattern 1=>g_*Ruminococcus*=>T5 Fine motor ability | 274 | -0.519 | -0.209 | 0.108 | 86% |
| Pattern 2_vs Pattern 1=>g_*Ruminococcus*=>T5 Personal-social ability | 274 | -0.547 | -0.218 | 0.135 | 88% |
| Pattern 3_vs Pattern 1=>g_*Ruminococcus*=>T5 Personal-social ability | 274 | -0.519 | -0.218 | 0.206 | 91% |

In analysis1-2, Women in pattern 1 experienced the highest air pollution with the highest psychological distress; women in pattern 2 experienced low pollution with moderate psychological distress; and women in pattern 3 experienced high pollution with low psychological distress.

In analysis3-4, women in pattern 1 experienced moderate air pollution with the highest psychological distress, women in pattern 2 experienced the highest pollution with low psychological distress, and women in pattern 3 experienced low pollution with moderate psychological distress.

In analysis5-6, women in pattern 1 experienced high air pollution fluctuations with low psychological distress fluctuations, and women in pattern 2 experienced low air pollution fluctuations with high psychological distress fluctuations.

In the analysis7-8, women in pattern 1 experienced low air pollution with low psychological distress, women in pattern 2 experienced moderate air pollution with high psychological distress, and women in pattern 3 experienced high air pollution with moderate psychological distress.


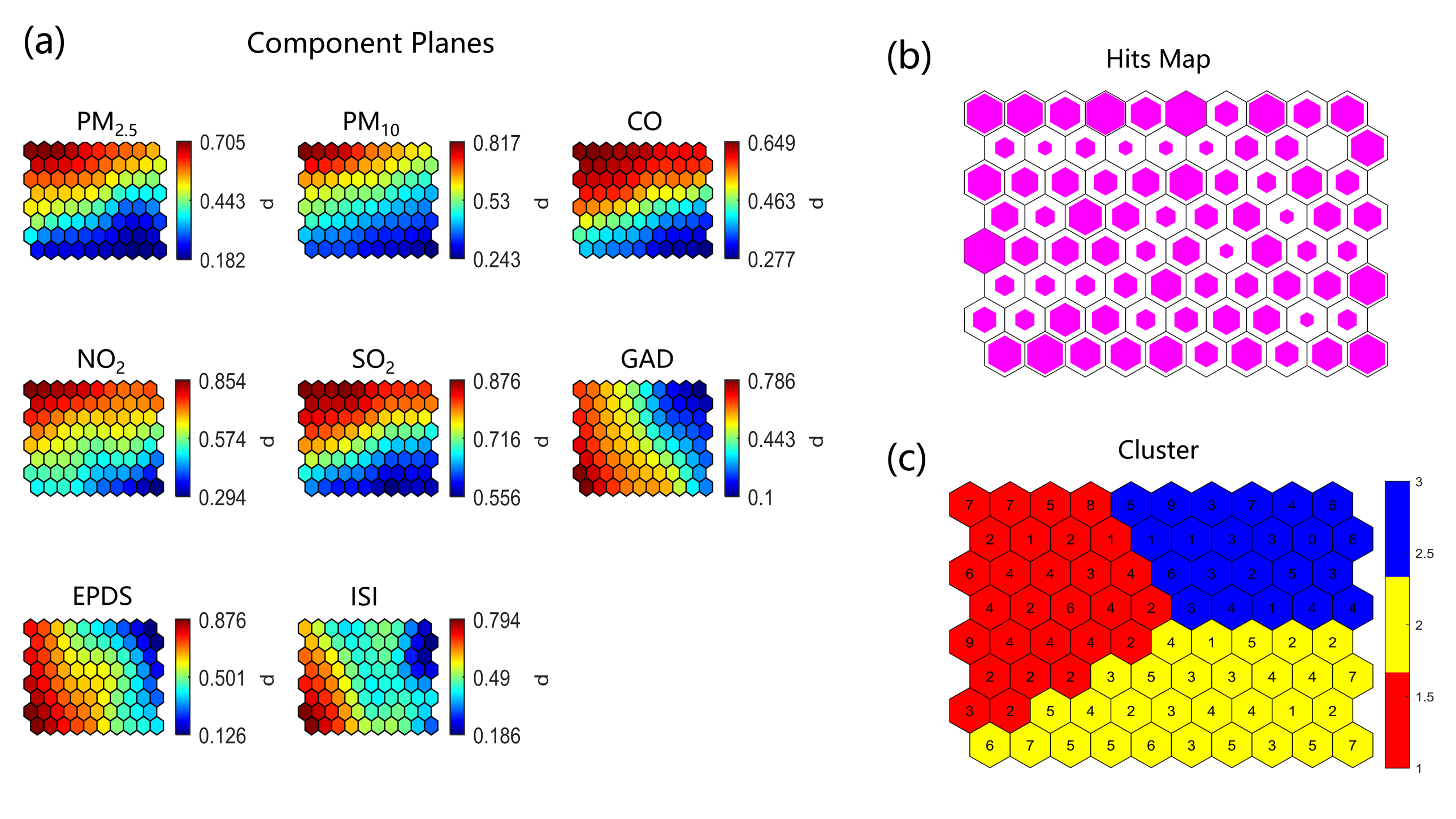


Figure S1. SOM results

(a) Component planes visualize the distribution of each type of maternal prenatal air pollution and psychological distress; (b) Hit map shows the neuron positions to which each sample is mapped, with the larger purple area indicating that the neuron contains more samples; (c) Pattern diagram represents the mapping and patterning of SOM samples. The grid and color represent an 8 × 10 matrix of neurons and the degree of similarity between neurons, respectively.


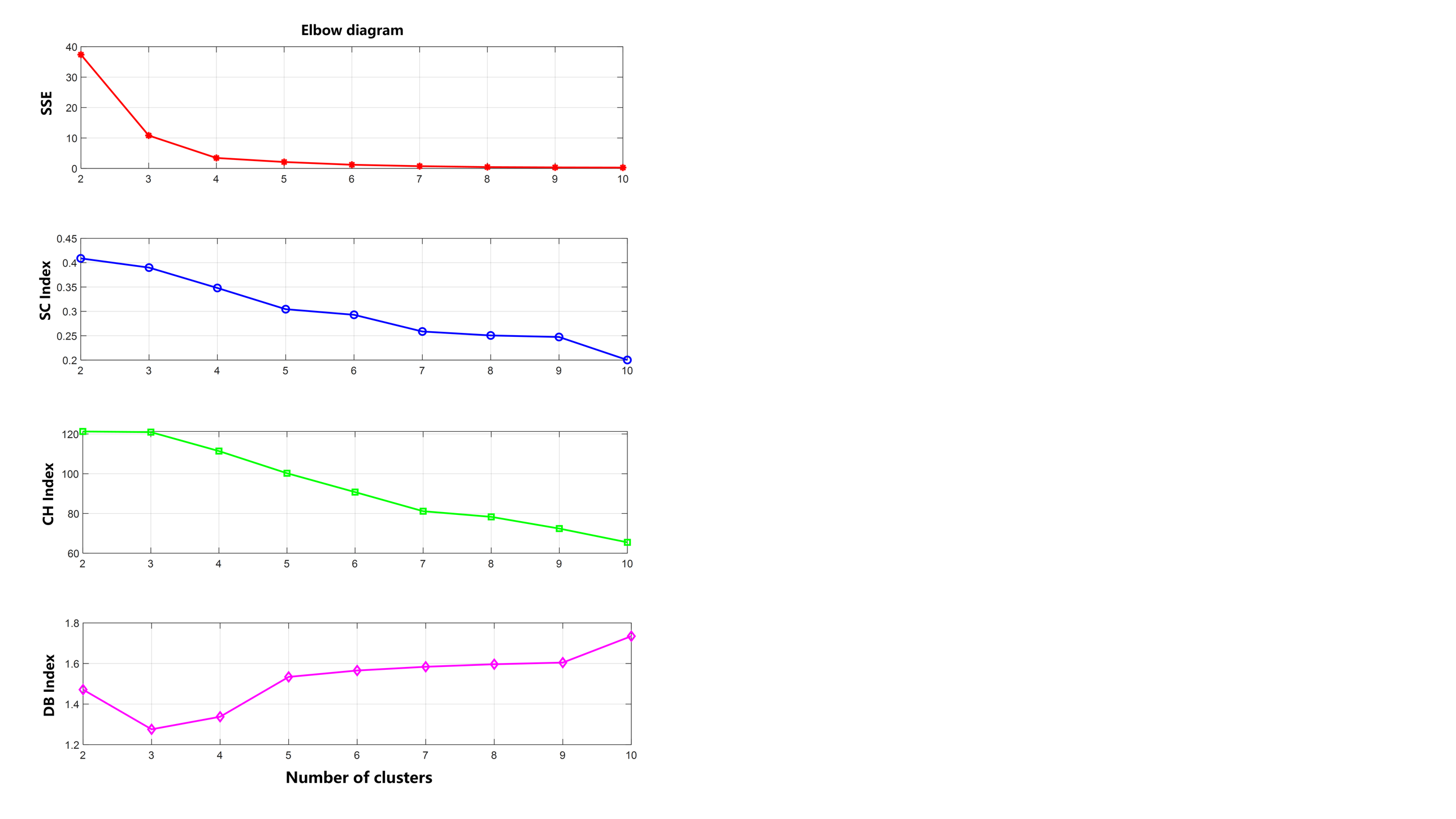


Figure S2. Parameters for selecting the number of clusters

SSE: Sum of Squared Errors, SC Index: Silhouette Coefficient Index, CH Index: Calinski-Harabasz index, and DB Index: Davies-Bouldin index.


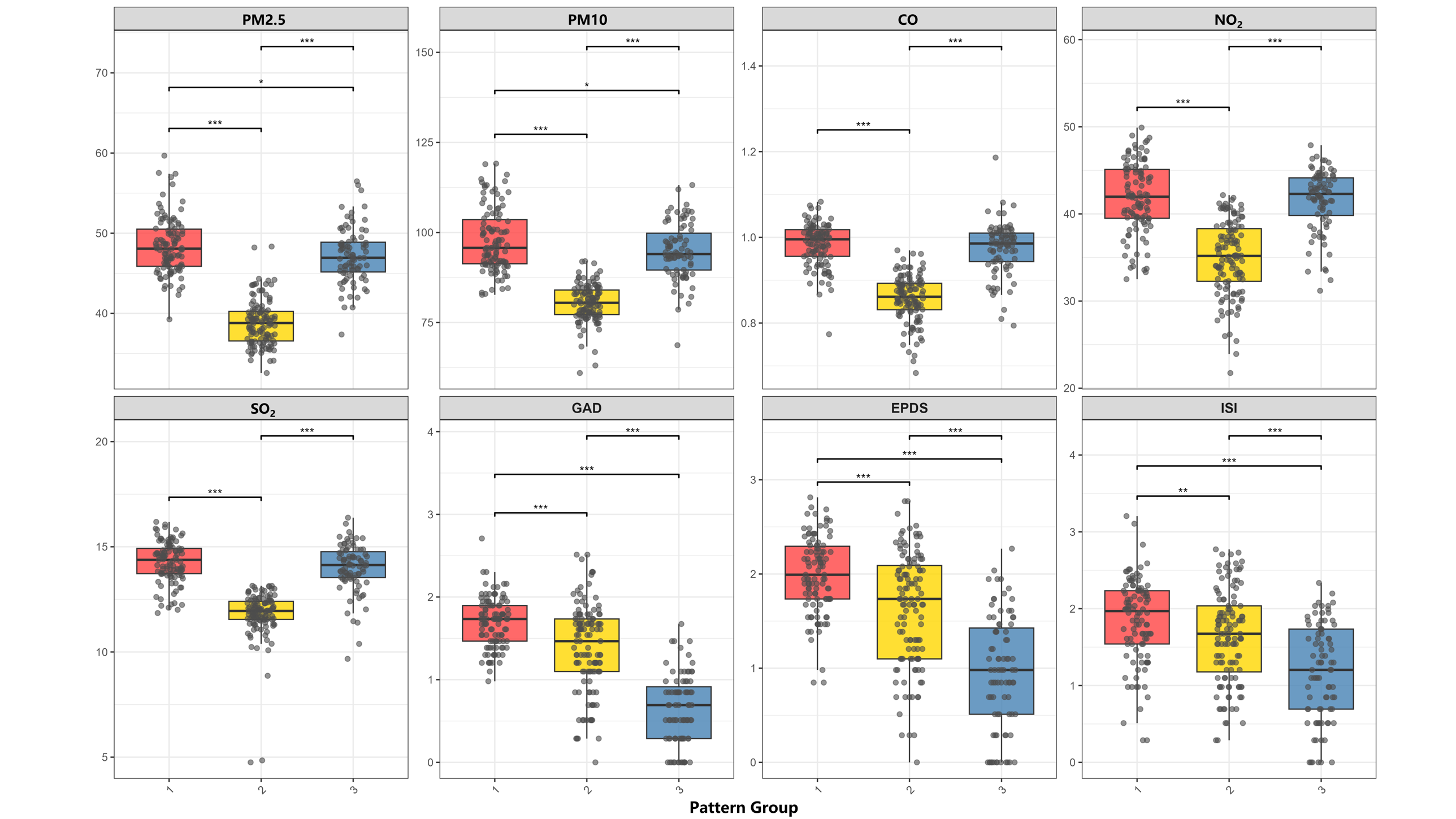


Figure S3. The inter-pattern comparison of maternal prenatal air pollution and psychological distress variables among the three patterns

* < 0.05; ** < 0.01; *** < 0.001.


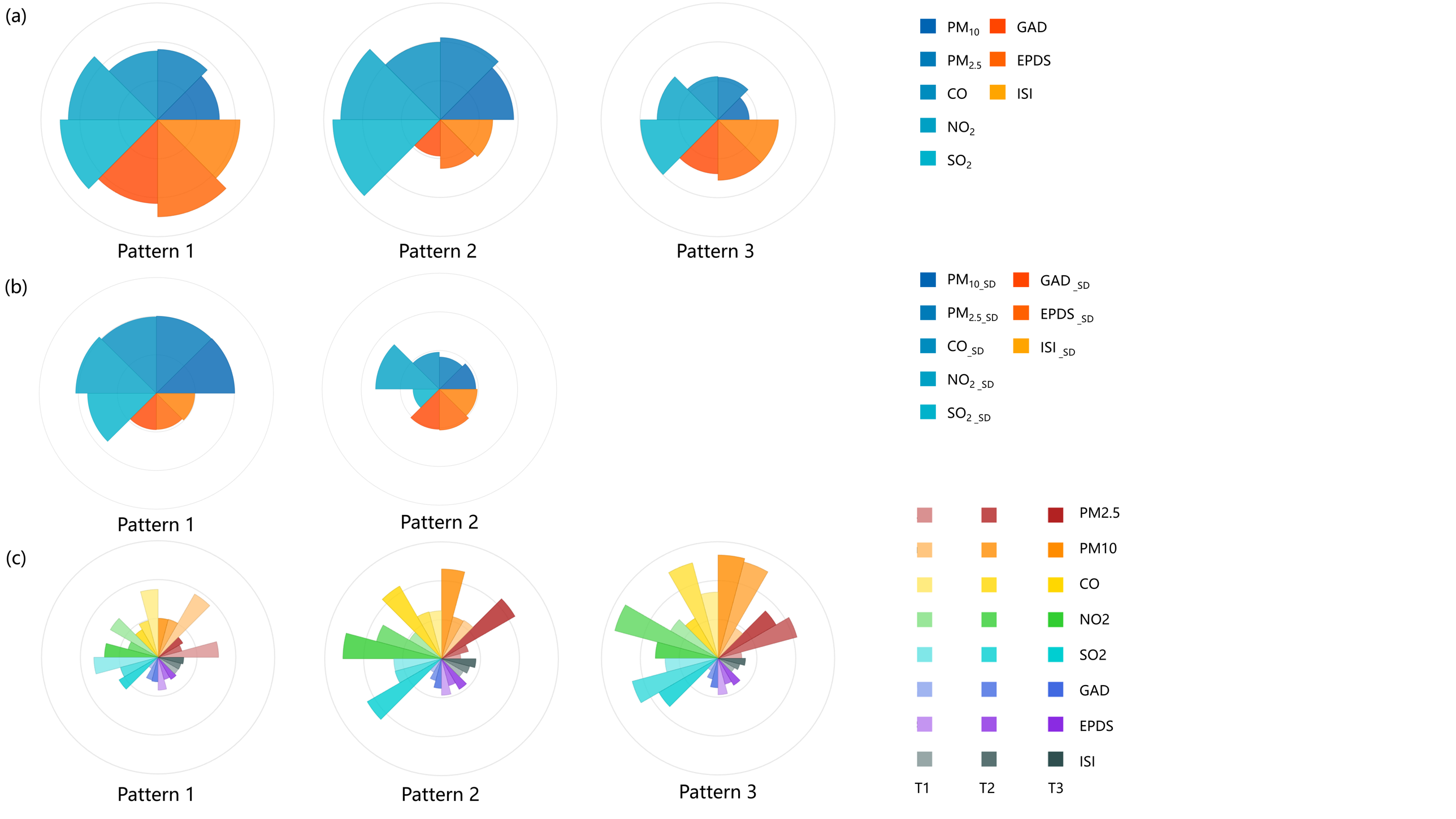


Figure S4. Nightingale rose diagram for the visualization of maternal prenatal co-exposure patterns

(a) After excluding mothers with complications during pregnancy, maternal prenatal co-exposure patterns were formed by the average. Women in pattern 1 experienced moderate air pollution with the highest psychological distress; women in pattern 2 experienced the highest pollution with low psychological distress; and women in pattern 3 experienced low pollution with moderate psychological distress. Women in pattern 3 were used as the reference group.

(b) Maternal prenatal co-exposure patterns formed using standard deviation. Women in pattern 1 experienced high air pollution fluctuations with low psychological distress fluctuations, and women in pattern 2 experienced low air pollution fluctuations with high psychological distress fluctuations.

(c) Maternal prenatal co-exposure patterns formed by 24 variables at three time points. The women in pattern 1 experienced low air pollution with low psychological distress, women in pattern 2 experienced moderate air pollution with high psychological distress, and women in pattern 3 experienced high air pollution with moderate psychological distress. Women in pattern 1 were used as the reference group.





Figure S5. Sensitivity analysis

a-d: Mediation analysis was conducted using the exposure patterns formed by the average while reducing the number of covariates.

e-g: Participants with pregnancy-related complications were excluded from the mediation analysis.

h-j: Participants with pregnancy-related complications were excluded from the mediation analysis, and the number of covariates was reduced.





Figure S6. Additional analysis

a-d: Conducting mediation analysis using the exposure patterns formed by standard deviation.

e-h: Conducting mediation analysis using the exposure patterns formed by standard deviation while reducing the number of covariates.
i-k: Conducting mediation analysis using the exposure patterns formed by 24 variables at three time points.

l-n: Conducting mediation analysis using the exposure patterns formed by 24 variables at three time points while reducing the number of covariates.


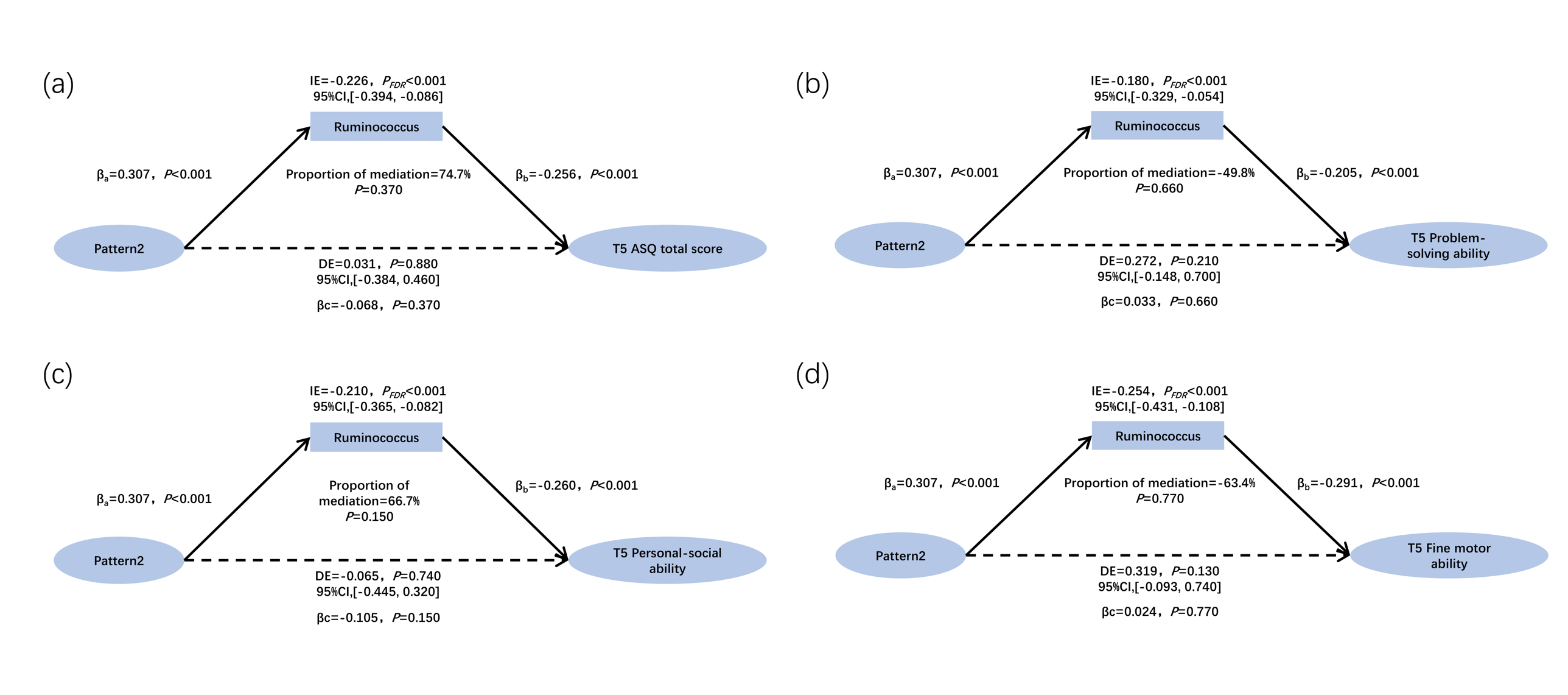


Figure S7. The mediating role of meconium microbiota in maternal prenatal co-exposure patterns and the infant neurodevelopment, pattern 3 was set as the reference

In the infant neurodevelopment model, we adjusted for maternal age, maternal education level, average monthly household income, pre-pregnancy body mass index, weight gain during pregnancy, complications during pregnancy, smoking during pregnancy, drinking during pregnancy, infant sex, delivery mode, infant gestational age at birth, infant birth weight, group B streptococci, vaginitis, frequency of intake of vegetables and fruits, frequency of intake of high-quality protein, and fetal feces collection time.


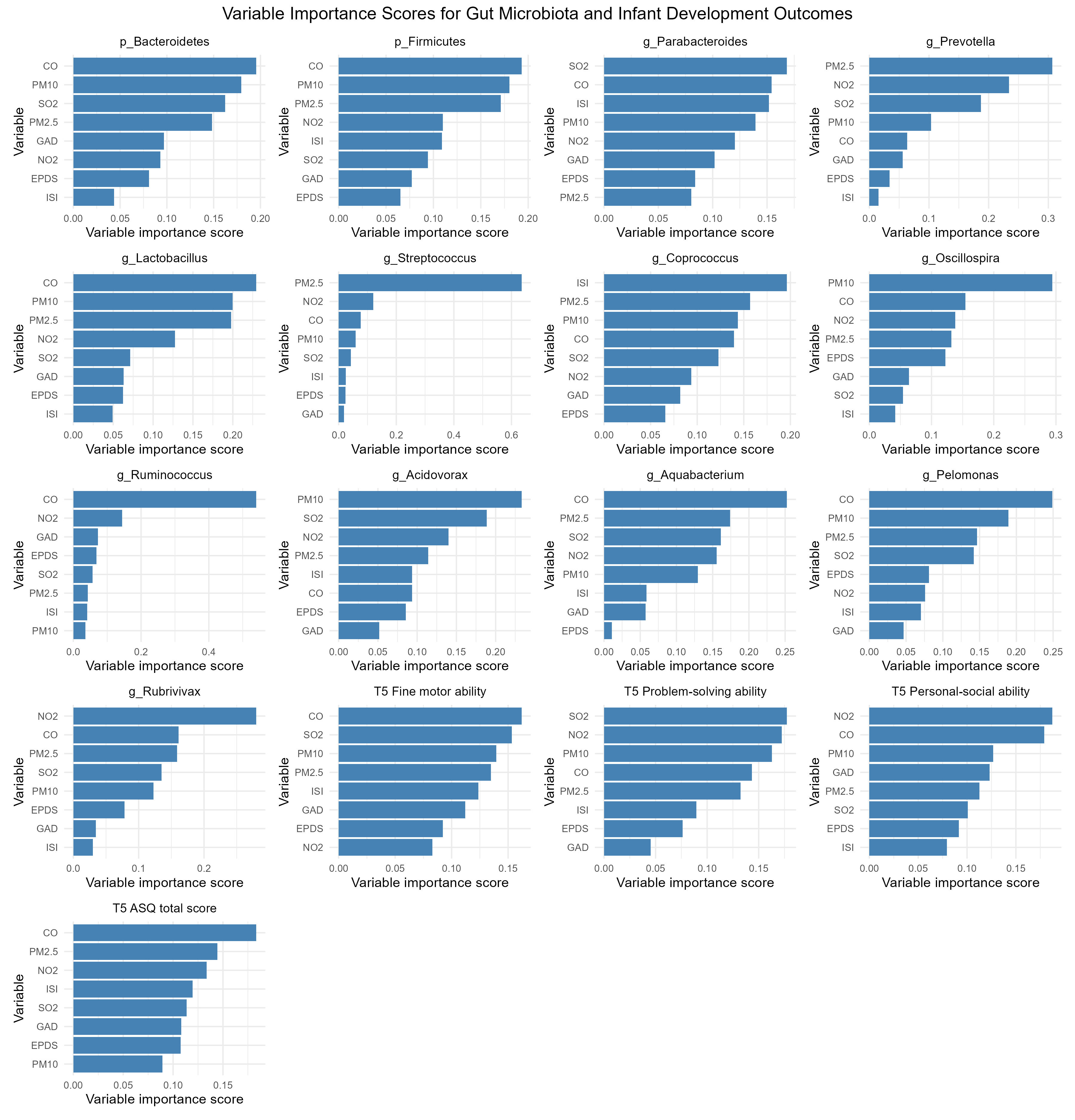


Figure S8. The importance ranking of 5 air pollution indicators and 3 psychological distress indicators for the meconium microbiota and infant neurodevelopment

The meconium microbiota taxa selected for analysis were those exhibiting significant differences in the MaAsLin results, excluding undefined or unclassified taxa. Infant neurodevelopmental indicators were selected from the indicators that were significant in the mediation analysis.


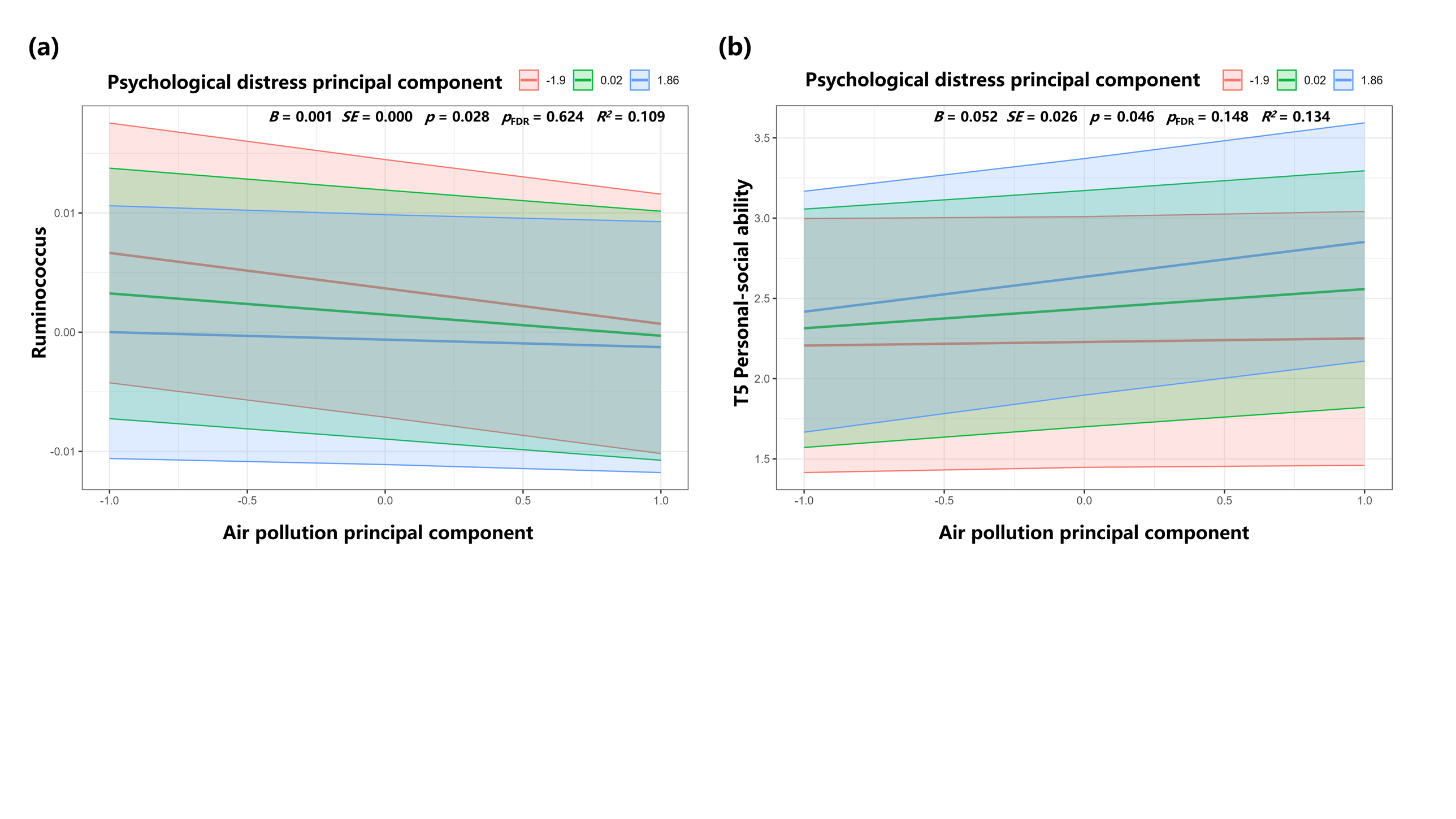


Figure S9. The interaction between maternal prenatal air pollution and psychological distress principal components on meconium microbiota and infant neurodevelopment

The infant neurodevelopment model was adjusted for maternal age, maternal education level, average monthly household income, pre-pregnancy body mass index, weight gain during pregnancy, complications during pregnancy, smoking during pregnancy, drinking during pregnancy, infant sex, delivery mode, infant gestational age at birth, and infant birth weight.

The meconium microbiota model was adjusted for additional variables, including group B streptococci, vaginitis, frequency of intake of vegetables and fruits, frequency of intake of high-quality protein, and fetal feces collection time.





Figure S10. Joint effect of the maternal prenatal co-exposure on infant neurodevelopment total scores at three time points and the significant meconium microbiota in the mediating analysis

a,d,g,j: Estimated weights of individual maternal prenatal co-exposure in the associations with ASQ total scores (T4, T5, and T6, respectively) and *Ruminococcus* in qgcomp analysis.

b,e,h,k: The overall effect of maternal prenatal co-exposure (estimates and 95%CI) on ASQ total scores (T4, T5, and T6, respectively) and *Ruminococcus* in the BKMR model.

c,f,i,l: Univariate exposure-response relationship of maternal prenatal co-exposure (estimates and 95%CI) with ASQ total scores (T4, T5, and T6, respectively) and *Ruminococcus* in the BKMR model.

T4, 1 months postpartum; T5, 3 months postpartum; T6, 6 months postpartum.

In the infant neurodevelopment model, we adjusted for maternal age, maternal education level, average monthly household income, pre-pregnancy body mass index, weight gain during pregnancy, complications during pregnancy, lifestyle during pregnancy, infant sex, delivery mode, infant gestational age at birth, and infant birth weight. In the meconium microbiota model, we adjusted for additional variables, including group B streptococci, vaginitis, frequency of intake of vegetables and fruits, frequency of intake of high-quality protein, and fetal feces collection time.
